# *UBTF* tandem duplications define a distinct subtype of adult de novo acute myeloid leukemia

**DOI:** 10.1038/s41375-023-01906-z

**Published:** 2023-04-21

**Authors:** Nicolas Duployez, Loïc Vasseur, Rathana Kim, Laëtitia Largeaud, Marie Passet, Anaïs L’Haridon, Pierre Lemaire, Laurène Fenwarth, Sandrine Geffroy, Nathalie Helevaut, Karine Celli‑Lebras, Lionel Adès, Delphine Lebon, Céline Berthon, Alice Marceau-Renaut, Meyling Cheok, Juliette Lambert, Christian Récher, Emmanuel Raffoux, Jean-Baptiste Micol, Arnaud Pigneux, Claude Gardin, Eric Delabesse, Jean Soulier, Mathilde Hunault, Hervé Dombret, Raphael Itzykson, Emmanuelle Clappier, Claude Preudhomme

**Affiliations:** 1 Université de Lille, Unité 1277-Canther, Institut National de la Santé et de la Recherche Médicale (INSERM), Lille, France; 2 Hematology Laboratory, Centre Hospitalier Universitaire (CHU) de Lille, Lille, France; 3 Université Paris Cité, Génomes, Biologie Cellulaire et Thérapeutique U944, INSERM, CNRS, F-75010 Paris, France; 4 Laboratoire de biologie médicale multisites SeqOIA – FMG2025, Paris, France; 5 Hematology Department, Saint Louis Hospital, AP-HP, Paris, France; 6 Hematology Laboratory, Saint Louis Hospital, Assistance Publique-Hôpitaux de Paris (AP-HP), Paris, France; 7 Hematology Laboratory, CHU Toulouse, INSERM 1037, CNRS, Université Toulouse III-Paul Sabatier, Centre de Recherches en Cancérologie de Toulouse, Toulouse, France; 8 Coordination Office, Acute Leukemia French Association, Paris, France; 9 Hematology Department, CHU Amiens-Picardie, Amiens, France; 10 Hematology Department, Claude Huriez Hospital, CHU Lille, Lille, France; 11 Hematology Department, Versailles Hospital, University Versailles-Saint-Quentin-en-Yvelines, Le Chesnay, France; 12 Service d’Hématologie, CHU Toulouse, Institut Universitaire du Cancer de Toulouse Oncopole, Université Toulouse III Paul Sabatier, Toulouse, France; 13 Hematology Department, Gustave Roussy Institute, Villejuif, France; 14 Hematology Department, CHU de Bordeaux, Bordeaux, France; 15 Hematology Department, Avicenne Hospital, AP-HP, Bobigny, France; 16 Unité 3518, Saint-Louis Institute for Research, Université de Paris, Paris, France; 17 Hematology Department, Université d’Angers, Université de Nantes, CHU Angers, Inserm, CNRS, CRCI2NA, SFR ICAT, F‑49000 Angers, France; 18 Fédération Hospitalo-Universitaire, Grand-Ouest Acute Leukemia, Angers, France

**Keywords:** Acute myeloid leukaemia, Cancer genomics

## Abstract

Tandem duplications (TDs) of the *UBTF* gene have been recently described as a recurrent alteration in pediatric acute myeloid leukemia (AML). Here, by screening 1946 newly diagnosed adult AML, we found that *UBTF*-TDs occur in about 3% of patients aged 18–60 years, in a mutually exclusive pattern with other known AML subtype-defining alterations. The characteristics of 59 adults with *UBTF*-TD AML included young age (median 37 years), low bone marrow (BM) blast infiltration (median 25%), and high rates of *WT1* mutations (61%), *FLT3*-ITDs (51%) and trisomy 8 (29%). BM morphology frequently demonstrates dysmyelopoiesis albeit modulated by the co-occurrence of *FLT3*-ITD. *UBTF*-TD patients have lower complete remission (CR) rates (57% after 1 course and 76% after 2 courses of intensive chemotherapy [ICT]) than *UBTF*-wild-type patients. In patients enrolled in the ALFA-0702 study (*n* = 614 patients including 21 with *UBTF*-TD AML), the 3-year disease-free survival (DFS) and overall survival of *UBTF*-TD patients were 42.9% (95%CI: 23.4–78.5%) and 57.1% (95%CI: 39.5–82.8%) and did not significantly differ from those of ELN 2022 intermediate/adverse risk patients. Finally, the study of paired diagnosis and relapsed/refractory AML samples suggests that *WT1*-mutated clones are frequently selected under ICT. This study supports the recognition of *UBTF*-TD AML as a new AML entity in adults.

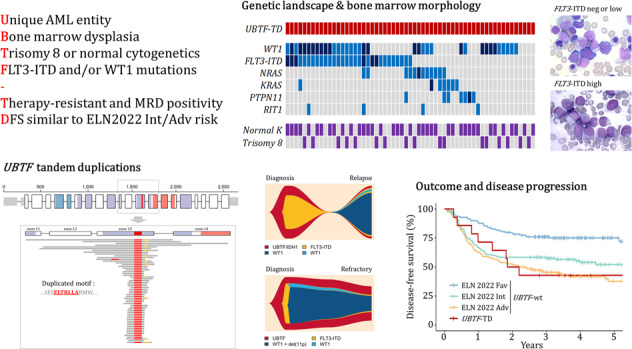

## Introduction

The upstream binding transcription factor (*UBTF*) gene, located at 17q21, encodes a ubiquitously expressed nucleolar protein. UBTF is a key component of the pre-initiation complex mediating the recruitment of RNA polymerase I to ribosomal DNA promoter regions but is also enriched at polymerase II-transcribed genes throughout human genomes [1–3]. UBTF is a member of the high mobility group (HMG)-box protein family, which contains six conserved HMG box DNA binding domains [4, 5]. *UBTF* dysregulation have been linked to different diseases including childhood neurodegeneration (due to germline gain-of-function missense mutations) [6] and cancers through various mechanisms. These include *UBTF* upregulation in solid tumors such as melanoma [7], lung [8] and colon cancers [9], or the oncogenic gene fusions *UBTF::ETV4* in prostate cancer [10] and *UBTF::ATXN7L3* in B-cell precursor acute lymphoblastic leukemia [11].

Recently, tandem duplications of *UBTF* (*UBTF*-TDs) have been described as a recurrent alteration in pediatric acute myeloid leukemia (AML), accounting for 4% of newly-diagnosed cases and 9% of relapse cases [12–15]. In pediatric AML, *UBTF*-TDs are associated with distinct genetic features including the frequent co-occurrence of *FLT3*-internal tandem duplications (*FLT3*-ITDs) and *WT1* mutations, normal karyotype or isolated trisomy 8, mutual exclusivity with known AML subtype-defining lesions (*i.e. NPM1* mutations and recurrent fusions) and activation of the *HOXA/HOXB* cluster genes [13]. *UBTF*-TDs were associated with poor outcome and measurable residual disease (MRD) positivity after induction chemotherapy. Together, these data suggest that *UBTF*-TD defines a new entity of high-risk pediatric AML [12–14, 16].

Here, we studied the prevalence of *UBTF*-TD in adult AML by screening 1946 newly diagnosed AML from 3 prospective trials. We describe the clinical, biological, genetic, and prognostic features associated with this alteration in a cohort of 59 AML patients with *UBTF*-TD (including 21 with prospectively collected survival data in the ALFA-0702 study) receiving intensive chemotherapy (ICT) usually followed by hematopoietic stem cell transplantation (allo-HCT).

## Methods

### Patients and samples

A total of 1946 available AML diagnostic samples from patients registered in 3 French ICT prospective trials were retrospectively screened for *UBTF-TD*. DNA samples were stored in the Tumor bank of the Acute Leukemia French Association (ALFA) in Lille Hospital (certification NF 96900-2014/65453-1). Patients were aged 18 to 60 years old in the ALFA-0702 (*n* = 614; NCT00932412) [17] and BIG1 (*n* = 895; NCT02416388) trials, and 60 years or older in the ALFA-1200 trial (*n* = 437; NCT01966497) [18].

Thirteen additional patients with *UBTF*-TDs identified in 2 centers from routine sequencing of AML samples (Saint-Louis Hospital AP-HP and CHU Lille) were also included to provide further insights into the disease description (Supplementary Fig. 1). All of them were registered in the Hauts-de-France (HDF)-AML observatory (Commission Nationale de l’Informatique et des Libertés identifier 2214454v0) and/or ALFA-PPP registry (NCT04777916). The study was conducted in accordance with the Declaration of Helsinki and French ethics regulations.

### UBTF-TD screening

Screening for *UBTF*-TD was performed by polymerase chain reaction (PCR) on genomic DNA extracted from bone marrow (BM) or peripheral blood (PB) using 6-FAM-labeled primers designed on exons 12 and 14 (Supplementary Table 1) of the *UBTF* gene. The Expand™ Long Template PCR System (Roche) with a touchdown PCR program (start from 65 °C to 62 °C with a decrease of 0.5 °C per cycle, followed by 28 cycles at 62 °C) was used because of off-target priming within the *UBTFL8* (*UBTF*-like 8) pseudogene in standard conditions. Then, PCR was followed by high resolution fragment analysis (4 h migration) on an Applied Biosystems™ 3730 DNA Analyzer 48-capillary array (ThermoFisher) using the GeneScan™ 1200 LIZ™ dye Size Standard (ThermoFisher).

### Targeted DNA sequencing

All samples with positive screening were further studied by captured-based next generation sequencing (NGS) with a custom panel of 154 genes including *UBTF* (Supplementary Table 2). Details about library preparation, sequencing and annotations are provided in the Supplemental Appendix.

### WGS, WES and RNA-sequencing

Whole genome sequencing (WGS), whole exome sequencing (WES) and whole transcriptome RNA sequencing (WTS) were performed in one patient (#L220L8879S) with refractory AML. Details are provided in the Supplemental Appendix.

### Statistical analysis

Variables are reported as numbers and percentages or median and interquartile range (IQR). Comparisons of categorical and continuous variables were made with Fisher exact and Mann-Whitney U tests, respectively. *P*-values were corrected for multiple testing by using the Benjamini-Hochberg procedure (*q*-values) [19]. As baseline features and outcome for *UBTF*-wt patients enrolled in the BIG1 trial were not available at time of this work, *UBTF*-TD patients were compared to the 593 *UBTF*-wt patients from the ALFA-0702 study only. Considering the rarity of *UBTF*-TDs in AML patients over 60 years, comparisons were done between patients aged 18 to 60 years only. Complete remission (CR)/CR with incomplete platelet recovery (CRp) rates were compared using the Fisher exact test. Multivariate analysis of CR/CRp was done with logistic regressions accounting for age, white blood cell (WBC) count and European LeukemiaNet (ELN) 2022 risk stratification [20]. Overall survival (OS) and OS from CR/CRp were defined from inclusion in the ALFA-0702 trial or time of CR/CRp achievement, respectively, until death or last follow-up. Disease-free survival (DFS) was defined from the date of CR/CRp to the date of relapse or death (whichever came first) or until last follow-up. Follow-up was estimated by the reverse Kaplan–Meier method. Survival was analyzed with the Kaplan–Meier method. Impact of *UBTF*-TD was estimated by the log-rank test for OS and DFS. The impact of allo-HCT on DFS and OS from CR/CRp was studied considering allo-HCT as a time-dependent variable in a univariable Cox model. All tests were two-sided, statistical significance was defined as a *p*-value or *q*-value <0.05, and statistical analyses were performed with R software 4.1.2 (cran.r-project.org).

## Results

### Prevalence of UBTF-TD and molecular characteristics

**Fig. 1:**
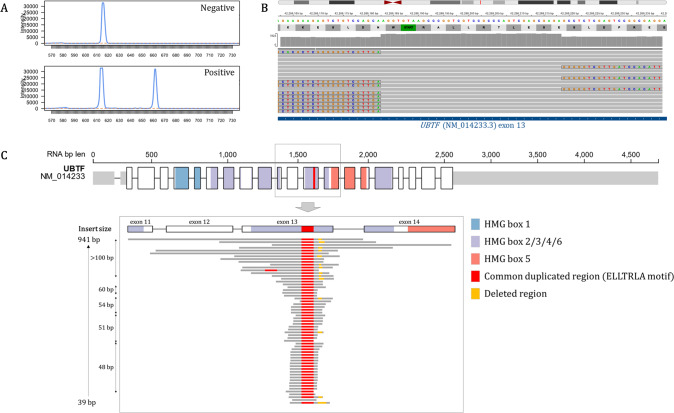
*UBTF*-TDs in adult AML. **A** Fragment analysis of mutant (665 bp) and wild-type (617 bp) alleles of *UBTF* in a patient with a 48-bp tandem duplication. **B** Integrative Genomics Viewer (IGV) visualization showing the soft-clipped reads and increased coverage in *UBTF* exon 13. **C** *UBTF* gene structure and location of *UBTF*-TDs. The common minimal duplicated motif in exon 13 is highlighted in red.

Presence of *UBTF*-TD was screened by fragment analysis and confirmed by targeted NGS (Fig. 1A–B) in 1946 patients with AML enrolled in 3 ICT trials. Somatic *UBTF*-TDs were identified in 3% of AML patients aged 18–60 years (21/614 [3.4%] and 23/895 [2.6%] patients from the ALFA-0702 and BIG1 cohorts respectively) but only 0.5% of AML patients 60 years or older (2/437 patients in the ALFA-1200 cohort). Together with patients prospectively identified by NGS, we report here a total of 59 adult AML patients with somatic *UBTF*-TDs.

*UBTF*-TDs were highly variable in size, ranging from 39 to more than 900 nucleotides. Most frequent TDs were 48 (*n* = 18), 51 (*n* = 10) and 54 (*n* = 6) base pairs (bp) in size, accounting together for 58% of all *UBTF*-TDs. All TDs led to in-frame insertions within exon 13 of *UBTF* with a common minimal duplicated region of 27 nucleotides (corresponding to nucleotides 1306 to 1326, using the NM_014233.3 transcript version as reference) shared by all but one patient. At the amino-acid level, this region encodes the leucine-rich ELLTRLA motif (Glu^436^-Leu^437^-Leu^438^-Thr^439^-Arg^440^-Leu^441^-Ala^442^) of the HMG4 domain (Fig. 1C, Supplementary Table 3). In the last patient not sharing this motif, the mutation was a 42-bp insertion encoding another leucine rich motif (GLCLRFNQLDLDQA). It should be noted that the size of very large duplications was not necessarily a multiple of 3 but was assumed to lead to in-frame insertions after RNA splicing. This hypothesis was verified by WTS in one patient (#L220L8879S) who harbored a large duplication (598 bp) spanning exons 12 to 14 finally leading to an exon13-exon13 fused RNA transcript (Supplementary Fig. 2).

As previously noticed in pediatric cases [13], we observed that most cases did not harbor perfect duplications. Insertions of non-templated nucleotides and deletions were frequent alongside the duplication. Fifteen patients (25%) had small deletions (mainly involving Trp^445^, Asn^446^ and Asp^447^) within the duplication. This feature was more frequent in large TDs >100 bp (64% vs. 13% in TDs <100 bp). In addition, while the variant callers used in our study (Vardict, Mutect2) generally failed to correctly annotate *UBTF*-TDs larger than 80 bp, this mismatch-creating feature enhanced our ability to suspect *UBTF* alterations with standards NGS algorithms. In all samples screened as positive, the variant callers identified at least one mismatch in *UBTF* (in contrast to *UBTF*-wt samples; data not shown). Because of difficulties to assess properly VAF of large insertions, VAFs for *UBTF*-TDs were measured using mismatches and data coverage. Across the 59 diagnostic samples, the median VAF of *UBTF*-TD was 45% (IQR 37–48) suggesting it was an early/founding event in leukemogenesis.

### Baseline features of adult AML patients with UBTF-TDs

**Fig. 2:**
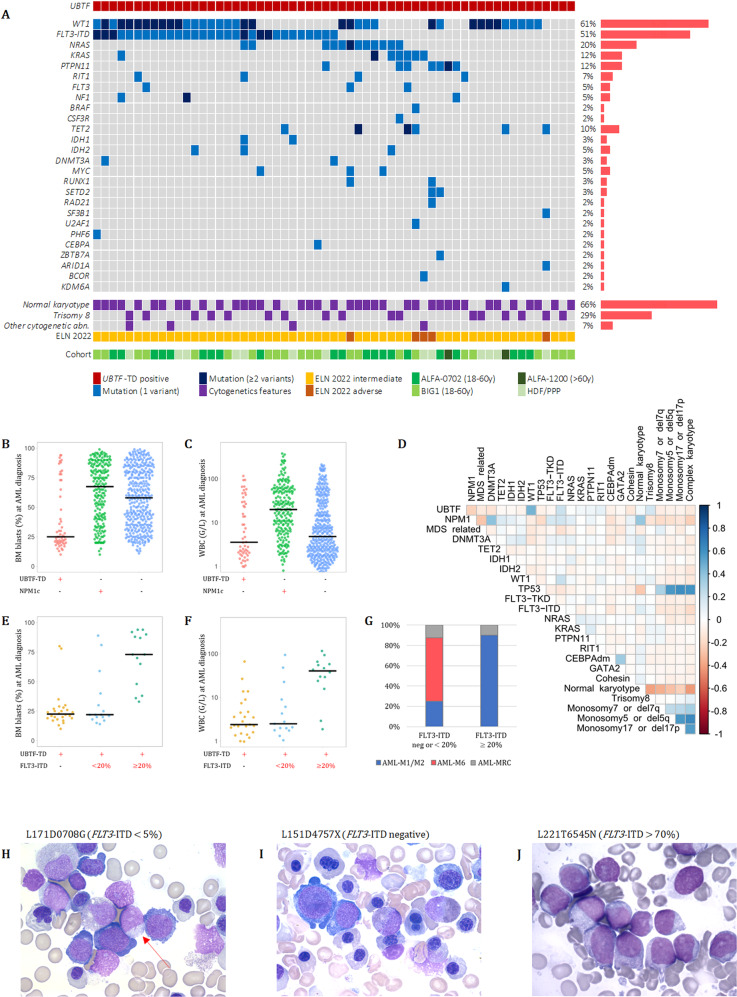
Baseline biological features associated with *UBTF*-TDs. **A** Mutational landscape of AML with *UBTF*-TD. Only genes with at least one mutation in one patient are shown. The full list of screened genes is given in the Supplementary Table 2. **B** BM blast infiltration and (**C**) WBC count according to *UBTF* and *NPM1* status in AML patients aged 18–60 years. The black bars indicate the median values. (**D**) Associations among gene and cytogenetic abnormalities in AML patients aged 18–60 years. (**E**) BM blast infiltration, (**F**) WBC count and (**G**) BM morphology classification according to *FLT3-ITD* status in *UBTF*-TD AML. The FiLT3r algorithm was used for detection and precise quantification of *FTL3*-ITDs [23]. **H** BM morphology in patient #L171D0708G (*FLT3*-ITD with VAF < 5%) and (**I**) patient #L151D4757X (*FLT3*-ITD negative) showing characteristic dyserythropoiesis and blasts with rare Auer rods (red arrow). **J** BM morphology in patient #L221T6545N (*FLT3*-ITD with VAF > 70%) showing massive blast infiltration.

Among 59 adult AML patients with somatic *UBTF*-TD, 39 were males (sex ratio 2/1). Median age at AML diagnosis was 37 years (IQR 23–47) and only 2 were over 60 years old. Median WBC count was 3.6 × 10^9^/L (IQR 2–26), and median BM blast infiltration was 25% (IQR 20–60) (Supplementary Table 4). Karyotype was normal in 39 patients (66%). The most frequent cytogenetic abnormality was trisomy 8 in 17 patients (29%, possibly found as a subclone). At least one additional mutation was found in 56 patients (95%) (median number of additional mutations: 3, range 0–9) (Fig. 2A). The most frequent co-occurring alterations were *WT1* mutations in 36 patients (61%) and *FLT3*-ITDs in 30 (51%). Twenty (33%) had both *WT1* and *FLT3*-ITD. Other mutations frequently involved signaling genes (*NRAS*, *KRAS*, *PTPN11*, *RIT1*), especially in cases lacking *FLT3*-ITD (55% of *FLT3*-ITD-negative patients presented at least one mutation in this set of genes). By contrast, mutations in epigenetic-related or RNA-splicing genes were rare. Finally, no other additional candidate gene was found by WGS/WES in patient #L220L8879S (Supplementary Table 5, Supplementary Table 6).

Among patients younger than 60 years, AML patients with *UBTF*-TD (*n* = 57) were significantly younger (median 36 years vs. 47 years; *q*-value <0.001), had lower BM blast infiltration (median 25% vs. 60%, *q*-value <0.001)and higher rates of *WT1* mutations (63% vs. 8%, *q*-value <0.001), *FLT3*-ITDs (53% vs. 22%, *q*-value <0.001) and trisomy 8 (28% vs. 8%, *q*-value <0.001) compared to *UBTF*-wt AML patients in the ALFA-0702 study (Fig. 2B–D, Table 1, Supplementary Table 7). Conversely, *UBTF*-TD was mutually exclusive with *NPM1* mutations, *CEBPA* in-frame bZIP mutations, *TP53* mutations, adverse cytogenetics and recurrent fusion transcripts and less frequently associated with myelodysplasia-related mutations [20] than *UBTF*-wt (9% vs. 29%, *q*-value = 0.003). The vast majority *UBTF*-TD AML were assigned to the ELN 2022 intermediate risk group. Only 5 were assigned to the adverse group because of myelodysplasia-related mutations (*RUNX1*, *n* = 2; *BCOR*, *n* = 1; *SF3B1*, *n* = 1; *U2AF1*, *n* = 1).

**Table 1:** Characteristics of AML patients (18–60 y) according to *UBTF* status.

Parameters	*UBTF*-TD AML 18–60 y	*UBTF* wild-type AML 18–60 y (ALFA-0702)
No. of patients	57	593
Age (y), median (IQR)	36 (24–45)	47 (37–54)
WBC (×10^9^/L), median (IQR)	3.55 (2–27.4)	8.3 (2.6–32.9)
BM blasts (%), median (IQR)	25 (20–63)	60 (39–82)
BM morphology, *n* (%)		
M0	0	28/467 (6%)
M1/M2	15/33 (45%)	254/467 (54%)
M4/M5	0	169/467 (36%)
M6	14/33 (42%)	14/467 (3%)
M7	0	2/467 (0%)
MRC	4/33 (12%)	
Cytogenetics		
Normal, *n* (%)	38/57 (67%)	329/563 (58%)
Trisomy 8, *n* (%)	16/57 (28%)	46/545 (8%)
Monosomy 5/del(5q), *n* (%)	0	33/545 (6%)
Monosomy 7/del(7q), *n* (%)	0	47/545 (9%)
Monosomy 17/del(17p), *n* (%)	0	22/545 (4%)
Del(20q), *n* (%)	0	14/545 (3%)
Del(12p), *n* (%)	0	14/545 (3%)
Complex, *n* (%)	0	67/545 (12%)
* WT1* mutations, *n* (%)	36/57 (63%)	48/572 (8%)
Signaling mutations		
* FLT3*-ITD, *n* (%)	30/57 (53%)	127/572 (22%)
* FLT3*-TKD, *n* (%)	4/57 (7%)	72/572 (13%)
* NRAS*, *n* (%)	13/57 (23%)	126/572 (22%)
* KRAS*, *n* (%)	7/57 (12%)	43/572 (8%)
* PTPN11*, *n* (%)	6/57 (11%)	32/572 (6%)
* RIT1*, *n* (%)	6/57 (11%)	19/572 (3%)
DNA-methylation gene mutations		
* DNMT3A*, *n* (%)	3/57 (5%)	162/572 (28%)
* TET2*, *n* (%)	5/57 (9%)	74/572 (13%)
* IDH1*, *n* (%)	3/57 (5%)	53/572 (9%)
* IDH2*, *n* (%)	3/57 (5%)	74/572 (13%)
*NPM1* mutations, *n* (%)	0	208/572 (36%)
*CEBPA* double mutations, *n* (%)	0	30/572 (5%)
*TP53* mutations, *n* (%)	0	39/572 (7%)
MDS-related gene mutations*, *n* (%)	5/57 (9%)	168/572 (29%)
ELN 2022 risk		
Favorable	0	143/548 (26%)
Intermediate	52/57 (91%)	157/548 (29%)
Adverse	5/57 (9%)	248/548 (45%)

*MDS-related genes: ASXL1, BCOR, EZH2, RUNX1, SF3B1, SRSF2, STAG2, U2AF1, and/or ZRSR2.

These data refer to only 57 of the 59 patients with *UBTF*-TDs (the 2 patients over 60 years of age were excluded for comparisons).

*BM* bone marrow, *IQR* interquartile range, *MRC* myelodysplastic-related changes, *WBC* white blood cell count.

BM morphology of *UBTF*-TD AML usually showed myelodysplastic features with trilineage dysmyelopoiesis including specific dyserythropoiesis (megaloblastosis, giant erythroblasts multinuclearity or nuclear lobulation) and megakaryocytic features (micromegakaryocytes). Blasts displayed myeloid features with myeloperoxidase positivity and rare Auer rods. Overall, 60% of cases showed a BM morphology compatible with the diagnosis of AML-M6 according to the FAB classification [21] or AML with myelodysplastic-related changes [22]. Interestingly, the co-occurrence of *FLT3*-ITD, especially at high VAF (≥20%), was associated with a specific AML presentation with higher WBC counts, massive BM blast infiltrations with disappearance of more mature cells and a dominance of the AML-M1/M2 morphological subtypes (Fig. 2E–J, Supplementary Fig. 3, Supplementary Table 8) [23].

### Disease history and evolution in UBTF-TD AML

Fifty-eight of the 59 patients with *UBTF*-TD AML received cytarabine-anthracycline based intensive induction chemotherapy (without *FLT3* inhibitors). Twenty-five (43%) failed to achieve CR/CRp after the first induction course among whom 10 finally achieved CR/CRp after a second salvage induction. This led to an overall CR rate of 57% after 1 course and 76% after 2 courses of ICT. The quantification of *WT1* transcript was performed at diagnosis and at early evaluation for 31 patients [24, 25]. As expected, *WT1* remained overexpressed after induction in all tested patients with refractory AML (*n* = 12). Among 19 patients in CR after the first induction course, 9 (47%) still had persistent *WT1* overexpression (Supplementary Fig. 4).

**Fig. 3:**
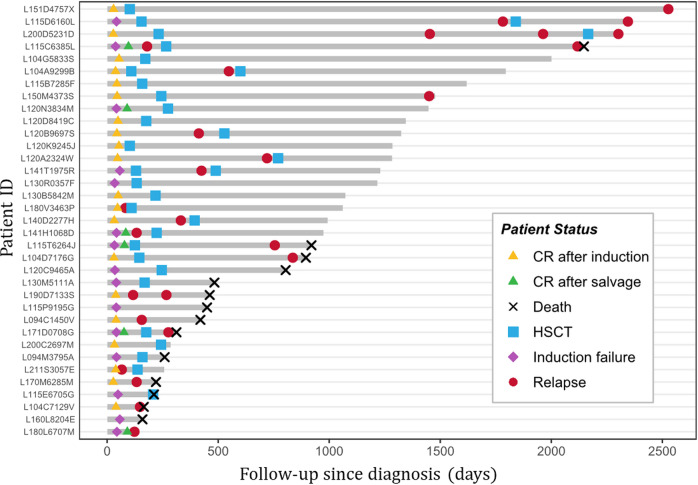
Swimmer plot graph of adult AML patients with *UBTF*-TDs. Each bar represents one patient in the study. Only patients with available follow-up (*n* = 35) are shown.

Figure 3 shows the disease history in 35 patients with available follow-up. Among those 35 patients, with a median follow-up of 2.7 years, 21 relapsed including 9 after allo-HCT and 12 before any allo-HCT. Median time from CR achievement to relapse in patients without allo-HCT was 3 months (range 1.2–21.6). The median time from allo-HCT to post-allo-HCT relapse was 22.8 months (range 3.6–79.2).

### Impact of UBTF-TD on CR achievement in AML patients 18–60 y treated with ICT

To explore the clinical impact of *UBTF*-TD on outcome, we compared the 21 *UBTF*-TD patients (all assigned to the ELN 2022 intermediate risk group) to the 593 *UBTF*-wt patients from the ALFA-0702 study. Among these 614 patients of the ALFA-0702 trial, 522 (85.0%) achieved CR/CRp after 1 or 2 courses of ICT. The proportion of *UBTF*-TD patients obtaining CR/CRp after 2 courses was 66.7% (*n* = 14) compared to 85.7% (*n* = 508) in *UBTF*-wt patients (*p*-value = 0.026) and 87.9% (*n* = 138) in ELN 2022 intermediate *UBTF*-wt patients (*p*-value = 0.018).

In multivariable logistic regression, *UBTF*-TD predicted lower rates of CR/CRp (OR = 0.23, 95%CI: 0.08–0.70, *p*-value = 0.007) independently of ELN 2022 risk stratification, WBC count and age (Table 2). *UBTF*-TD still predicted lower rates of CR/CRp (OR = 0.27, 95%CI: 0.08–0.88, *p*-value = 0.027) when restricted the analysis to patients in the ELN 2022 intermediate risk group, independently of WBC count and age. While it represents a small number of patients, among *UBTF*-TD patients enrolled in the ALFA-0702 study, *WT1* mutations were associated with lower CR/CRp rates (*n* = 4/10 [40%] vs. *n* = 10/11 [91%], *p*-value = 0.024). Conversely, among *WT1*-mutated patients in the ALFA-0702 study, *UBTF*-TD was associated with lower CR/CRp rates (*n* = 4/10 [40%] vs. *n* = 38/48 [79%], *p*-value = 0.020).

**Table 2:** Multivariate logistic regression for CR/CRp achievement in ALFA-0702 cohort.

	CR/CRp OR (95%CI)	*p*-value
*UBTF* wild-type	1	
*UBTF*-TD	0.23 (0.08–0.70)	0.007
Age (per 10-years of age)	0.96 (0.77–1.18)	0.69
Log10(WBC)	0.69 (0.47–1.01)	0.06
ELN 2022 Intermediate risk	1	
ELN 2022 Favorable risk	3.64 (1.41–11.26)	0.01
ELN 2022 Adverse risk	0.37 (0.20–0.67)	<0.0001

### Prognostic significance of UBTF-TD in AML patients 18–60 y treated with ICT

**Fig. 4:**
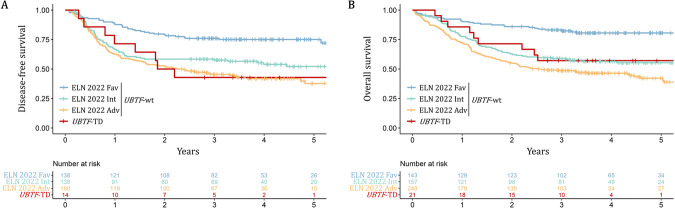
Clinical outcome of *UBTF*-TD AML 18–60 y. **A** Disease-free survival in patients achieving CR or CRp after induction and (**B**) overall survival according to *UBTF* status and ELN 2022 risk stratification. Study restricted to patients enrolled in the ALFA-0702 trial with available ELN 2022 risk stratification.

We next studied the prognostic value of *UBTF*-TD on DFS and OS in the ALFA-0702 cohort. Among 548 *UBTF-*wt patients for whom the ELN 2022 risk stratification was available, 26% were favorable (*n* = 143), 29% were intermediate (*n* = 157) and 45% were adverse (*n* = 248). The 3-year DFS and OS were 42.9% (95%CI: 23.4–78.5%) and 57.1% (95%CI: 39.5–82.8%) in *UBTF*-TD patients (*n* = 21) compared to 57.6% (95%CI: 53.4–62.1%; *p*-value = 0.5) and 60.9% (95%CI: 57.0–64.9%; *p*-value = 1.0) in *UBTF*-wt patients. When considering ELN 2022 risk stratification, DFS of *UBTF*-TD patients was significantly lower than DFS in *UBTF*-wt favorable risk patients (3y-DFS: 76.0%, 95%CI: 69.2–83.5%, *p*-value = 0.008) but not significantly different from DFS in *UBTF*-wt intermediate (3y-DFS: 58.4%, 95%CI: 50.7–67.3%, *p*-value = 0.6) and adverse risk patients (3y-DFS: 45.4%, 95%CI: 38.8–53.1%, *p*-value = 0.8). Similarly, OS of *UBTF*-TD patients was lower than OS in *UBTF*-wt favorable risk patients (3y-OS: 82.3%, 95%CI: 76.3–88.9%, *p*-value = 0.01) but not significantly different from OS in *UBTF*-wt intermediate (3y-OS: 59.0%, 95%CI: 51.7–67.2%, *p*-value = 0.9) and adverse risk patients (3y-OS: 48.7%, 95%CI: 42.8–55.3%, *p*-value = 0.3)(Fig. 4A–B). Furthermore, although this only refers to a limited number of patients, the OS of *UBTF*-TD AML tended to worsen with the cooccurrence of *WT1* mutations (3y-OS: 20% [95%CI: 6–69%] vs. 91% [95%CI: 75–100%], *p*-value = 0.002) or *FLT3*-ITD (3y-OS: 25% [95%CI: 8–83%] vs. 77% [95%CI: 57–100%], *p*-value = 0.008)(Supplementary Fig. 5).

According to the design of the ALFA-0702 study, all *UBTF*-TD patients (*n* = 21) were assigned to the intermediate risk group, and were thus eligible to allo-HCT. Among 14 *UBTF*-TD patients who achieved CR/CRp, 9 underwent allo-HCT in first CR/CRp (64.3%) and 3 after relapse (21.4%). Among this limited number of patients, allo-HCT in first CR/CRp seemed to be associated with prolonged DFS (2y-DFS: 77.8% vs. 0%, *p* = 0.00976) but not with prolonged OS from CR/CRp (2y-OS 100% vs. 65.6%; *p* = 0.521) (Supplementary Fig. 6).

### Clonal architecture and disease evolution of AML with UBTF-TD

**Fig. 5:**
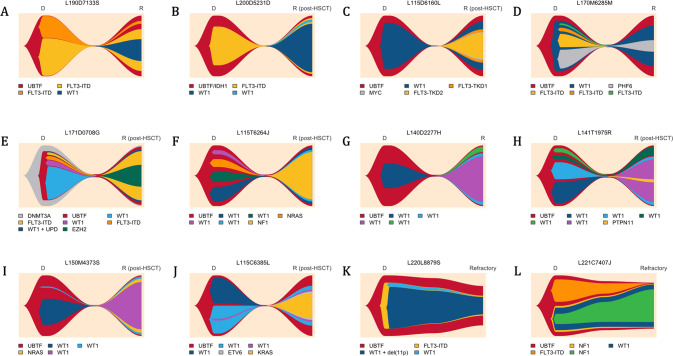
Patterns of clonal evolution in *UBTF*-TD AML. Fishplots are imputed from VAFs obtained by bulk NGS at AML diagnosis and during disease progression (relapsed or refractory AML). Twelve patients with available matched samples are presented: (**A**–**J**) Relapsed AML; (**K**–**L**) Refractory AML. Disappearance of all mutations (including *UBTF*-TD) in CR was verified by NGS (1% threshold) in patients **A**, **B**, **C**, and **G** and was assumed in the others. Details about patient history are provided in the Fig. 3. Figure made with the Fishplot package for R (version 4.2.0) [26].

To better understand the clonal architecture of *UBTF*-TD AML and investigate the co-occurrence and order of acquisition of somatic mutations, 12 cases were studied both at diagnosis and during evolution (relapsed AML, *n* = 10 or refractory AML, *n* = 2)(Fig. 5) [26]. The clonal evolution was inferred from bulk NGS data considering VAF as a surrogate measure of clonal abundance. VAFs were corrected with copy number analysis to account for biallelic alterations. Patient #L220L8879S was shown to carry both *WT1* mutation and deletion. Patient #L171D0708G was shown to carry a homozygous *WT1* mutation probably due to an acquired uniparental disomy [27]. In most cases, *UBTF*-TD was present with the highest VAF at both timepoints (except for patients #L171D0708G and #L200D5231D for whom *UBTF*-TD probably occurred after or concomitantly with *DNMT3A* and *IDH1* mutations). *WT1* and *FLT3*-ITD mutations appeared to involve only a subset of leukemic cells and were frequently subject to clonal evolution during progression, suggesting they are late cooperative events (Supplementary Fig. 7).

Analysis of paired diagnostic-relapse cases revealed a preferential pattern of disease progression likely driven by *WT1* mutations. All relapse AML cases (*n* = 10) harbored at least one *WT1* mutation with high VAF compatible with clonal dominance (Supplementary Fig. 8). Moreover, 6 of the 10 relapse AML cases carried multiple *WT1* mutants, with VAFs suggesting that 2 mutants coexisted in the same clone in 5 cases (patients referred as F to J in the Fig. 5 and Supplementary Fig. 8). Another one (patient E) was shown to carry a homozygous *WT1* mutation at relapse. Overall, biallelic *WT1* alterations (WT1^bi_alt^) were found in 6/10 (60%) relapse samples. The WT1^bi_alt^ clone driving relapse was already present at diagnosis at low levels in 2 cases (E and J), derived from a *WT1* monoallelic clone (WT1^mono_alt^) in 3 cases (F, H and I), or fully emerged at relapse (or was present below the sensitivity threshold [VAF 1%] at diagnosis) in 1 case (patient G). Other mutations could coexist within the WT1^bi_alt^ clone at relapse, frequently involving the RAS pathway (*NF1*, *PTPN11*, *KRAS*, and *NRAS* in patients F, H, I and J). In addition, patient K who was the only studied patient with a dominant WT1^bi_alt^ clone (mutation + deletion) at AML diagnosis was refractory to induction, suggesting the resistance of this clone to intensive chemotherapy. Consistent with these observations, 21/35 (60%) *WT1*-mutated patients (regardless of the number or VAF of the mutation) receiving ICT were refractory to the first course of ICT, compared with 4/23 (17%) *WT1* wild-type patients.

Another pattern of disease relapse was observed in patients A and B for whom the relapse appeared to be driven by a WT1^mono_alt^ arising in a preexisting *FLT3*-ITD clone. While *FLT3*-ITD is generally associated with resistance and disease progression in AML [28], *FLT3*-ITDs did not seem to confer clonal advantage in *UBTF*-TD AML when not associated with *WT1* mutation. Indeed, some *FLT3*-ITDs detected at diagnosis were frequently lost during progression (patients A, D and E). Similarly, patient L who was refractory to induction chemotherapy (without *FLT3* inhibitor) demonstrated the rapid disappearance of *FLT3*-ITD and the selection of a preexisting WT1^mono_alt^/*NF1*-comutated clone.

Finally, 2 patients in the cohort received a FLT3 inhibitor (FLT3i; quizartinib) at relapse, one of whom (patient A) experienced a second relapse that was documented by sequencing. The post-quizartinib relapse was still positive for *FLT3*-ITD and characterized by the emergence of 2 distinct *FLT3*-ITD/*FLT3*-TKD (D835H/Y) mutants, which have been described as a recurrent mechanism of resistance to FLT3 inhibitors (Supplementary Fig. 9).

## Discussion

Molecular and cytogenetic analyses are routinely used to identify AML subtype-defining structural variants and mutations and have become critical for risk stratification and treatment guidance. Despite 15 years of genomics research since the first AML genome publication and large studies like The Cancer Genome Atlas (TCGA), some AML cases remain genetically unclassifiable with current knowledge [29–31]. Notably, a subset of AMLs with *FLT3*-ITD and/or *WT1* mutation were known to lack a known initiating event, while being associated with a poor outcome in both adults and children [27, 32]. The aggregation of some of these AMLs under a single entity, namely *UBTF*-TD AML, paves the way for their detection in routine practice and future development of new therapies. Overall, it is likely that *UBTF*-TDs have been underestimated in previous studies due to limitations in detecting this type of aberration with many of the current bioinformatic approaches used in genetics laboratories [13]. This may be due to the difficulty to align tandem duplications-containing reads to the reference genome but also to the complexity of the *UBTF* sequence which includes repetitive motifs and frequent homopolymers complicating variant calling.

All TDs identified in the present study led to in-frame insertions within exon 13 of *UBTF* with a common minimal duplicated region encoding the ELLTRLA motif of the HMG4 domain of the UBTF protein, as previously described in pediatric cases [13]. TDs ranged from 39 to more than 900 nucleotides with frequent insertions of non-templated nucleotides and deletions alongside the duplication. This feature has been already observed in other in frame duplications such as *FLT3*-ITD [33]. The question of a common pathophysiologic background involved in the genesis of these 2 anomalies deserves to be explored. Also, the consequences of the exon 13 duplication on UBTF function and leukemogenesis are still unknown. Umeda et al have shown that *UBTF*-TD expression in CD34 + cells was sufficient to induce a proliferative advantage, increases clonogenic activity and activates the *HOXB* gene cluster, recapitulating the transcriptional signature observed in *UBTF*-TD AML patients [13].

Here we found that *UBTF*-TDs occur in about 3% of AML in patients aged 18–60 years. However, given the variability of TDs, we cannot exclude that some perfect and large duplications were missed by the screening method. Adult AML patients with *UBTF*-TDs were significantly younger and had lower BM blast infiltration with a BM morphology frequently demonstrating myelodysplastic features including severe dyserythropoiesis. It should be noted that *UBTF*-TD AML were associated with the M6 morphology subtype [34] according to the FAB classification (especially in cases without *FLT3*-ITD or low ratio) and the diagnosis of AML could thus be made by counting the proportion of BM blasts among the non-erythroid cells according to the FAB criteria [21]. It is likely that some patients may have been diagnosed with myelodysplastic syndrome with increased/excess blasts using current classifications [35, 36].

*UBTF*-TDs were mutually exclusive with other known AML subtype-defining alterations. Sequencing data revealed *UBTF*-TDs were always clonal and stable during disease progression, in agreement with an early/initiating event. The most frequent co-occurring somatic aberrations were by far *WT1* mutations, *FLT3*-ITD (high ratios being associated with increased WBC count and BM blast infiltration) and trisomy 8 (85% of cases carried at least one of these aberrations and 47% had at least two). Patients with *UBTF*-TDs displayed high rates of induction failure.

Prognostic analyses in patients enrolled in the ALFA-0702 study (including 21 *UBTF*-TD AML) showed that DFS and OS of *UBTF*-TD patients were not significantly different from those of *UBTF*-wt ELN 2022 intermediate or adverse risk patients. Although long-term remissions were observed after allo-HCT, post-allo-HCT relapse remained common and could occur after a significant delay. This shows that surveillance as well as MRD and chimerism monitoring should be repeated and would potentially allow for preemptive measures (*e.g*. donor lymphocyte infusion, immunomodulation). However, considering the limited number of patients studied, additional data are required to confirm the prognosis of *UBTF*-TD AMLs and assess the benefits of allo-HCT in this context.

Interestingly, the study of paired diagnosis and relapsed/refractory AML samples showed that *WT1* mutations are likely to play a major role in disease progression. Especially, WT1^bi_alt^ or WT1^mono_alt^/*FLT3*-ITD co-mutations were observed in most relapses, whereas patients with these features at AML diagnosis were more likely to be refractory to induction chemotherapy. Together, these results suggest strong cooperation within the *UBTF*-TD/*WT1*/*FLT3*-ITD trio in establishing the leukemic phenotype and determining the clinical outcome. Also, these results emphasize the need to evaluate new therapies in combination to first-line treatment or in maintenance. FLT3 inhibitors have been shown to improve outcome in *FLT3*-ITD-positive AML but it should be noted that *WT1* mutations have been identified as contributing to drug resistance [37, 38]. The mechanism of *WT1* mutations in leukemogenesis remains elusive. WT1 has a functional duality since it may act, depending on the cellular context, as a transcriptional activator or repressor through its DNA-binding zinc-finger domain and interactions with Tet enzymatic proteins that regulate DNA hydroxymethylation [39, 40]. Given this epigenetic role, the use of hypomethylating agents such as azacytidine should also be considered as a potential strategy in these cases [41]. Finally, given the high expression of *HOX* cluster genes in *UBTF*-TD AML, the use of menin inhibitors is also a promising therapeutic approach that deserves to be studied in the future [42].

Overall, our study revealed that *UBTF*-TDs can be found in about 3% of adult AML aged 18–60 years and define a distinct subtype of AML with specific biological and clinical features and lower CR rates. Together with pediatric data, this study supports the recognition of *UBTF*-TD AML as a new AML entity to be included in disease classifications, a prerequisite for the development of future guidelines and therapies, especially in young patients.

## Supplementary information

Supplemental appendix

Supplemental tables

## Data Availability

The datasets generated during and/or analysed during the current study are available from the corresponding author on reasonable request.
