# Supplementary material for: UBTF tandem duplications define a distinct subtype of adult de novo acute myeloid leukemia
Source: Leukemia. 2023 Apr 21;37(6):1245–53. doi: 10.1038/s41375-023-01906-z (PMC10244165; doi:10.1038/s41375-023-01906-z)
Supplement: Supplementary file 1 — Supplemental appendix [file 41375_2023_1906_MOESM1_ESM.pdf]

Supplemental data

UBTF tandem duplications define a distinct subtype of adult *de novo* acute myeloid leukemia

Nicolas Duployez et al.

SUPPLEMENTAL METHODS

Targeted DNA sequencing

All samples with positive screening were further studied by captured-based next generation sequencing (NGS) with a custom panel of 154 genes (**Supplementary Table 2**). Libraries were prepared according to the Twist® NGS target enrichment solution (Twist BioScience®) following the manufacturer's instructions and run on NovaSeq6000® (Illumina®) with a median read depth of 3600X. Raw NGS data were analyzed with MuTect2[1] and Vardict[2] for variant calling and the inhouse NGSreport Software (CHU Lille) for data visualization, elimination of sequencing/mapping errors and retention of variants with high quality metrics. The FiLT3r algorithm was used for detection and precise quantification of *FTL3*-ITDs.[3] Somatic copy number variant (CNV) analysis was performed following the "GATK Best Practices" (Broad Institute 2022) using coverage data and normalization with a panel of normal samples. Variant allele frequencies (VAFs) were measured as the number of sequence reads matching a specific DNA variant divided by the overall coverage at that locus. Variants were named according to the Human Genome Variation Society (GRCh37/hg19 build).[4] Variant interpretation was performed considering minor allele frequencies (MAF) in the public GnomAD database of polymorphisms (variants with MAF > 0.02 in overall population/global ancestry or sub-continental ancestry were excluded) and VAFs. Samples from patients diagnosed by routine NGS and relapse samples were processed with the same workflow. The clonal evolution schemes were imputed from bulk NGS data on diagnostic/relapse samples, considering VAF as a surrogate measure of clonal abundance, and designed using the Fishplot package for R (version 4.2.0).[5] Genomic annotations (including mutational analysis and fusion transcript detection) for *UBTF*-wild-type (wt) AML patients enrolled in the ALFA-0702 and ALFA-1200 trials were assessed centrally on genomic DNA, as previously published.[6,7]

WGS, WES and RNA-sequencing

Whole genome sequencing (WGS), whole exome sequencing (WES) and whole transcriptome RNA sequencing (WTS) were performed on BM relapse sample from patient #L220L8879S, at the SeqOIA laboratory (https://laboratoire-seqoia.fr/). WGS was also performed on PB purified $CD3^+$T-cells (considered as a germline material).

Production of WGS and WES sequences was performed as follow: Nucleic acids were fragmented by sonication (LE220plus®, Covaris®), size-selected and purified on magnetic beads (Sera-Mag magnetic beads®, GE Healthcare®). The preparation of the libraries (NEBNext® Ultra II End repair/A-tailing module & Ligation module, New England Biolabs®) were performed without amplification for WGS or with amplification of pre-capture libraries by PCR (KAPA Hifi HotStart ReadyMix, Roche®) for WES. Exome capture was performed by single-plex hybridization (Twist Human Core Exome Kit + IntegraGen Custom v1, Twist BioScience®) with amplification by PCR and the captured regions were amplified by PCR (KAPA Hifi HotStart ReadyMix, Roche®). WGS and WES libraries were sequenced in 'pair-end' (2×150 cycles) on NovaSeq 6000® (Illumina®).

Production of RNAseq sequences was performed as follow: A complementary DNA library was generated by reverse transcription of mRNAs after poly(A) capture (NEBNext® Poly(A) mRNA Magnetic Isolation Modules, NEBNext® Ultra II RNA First Strand Synthesis Module & Directional RNA Second Strand Module, New England Biolabs®). Size selection and subsequent purification steps were performed on magnetic beads (Sera-Mag magnetic beads®, GE Healthcare®). The library preparation (NEBNext® Ultra II End repair/A-tailing module & Ligation module, New England Biolabs®) was amplified by PCR (KAPA Hifi HotStart ReadyMix, Roche®). The library was sequenced in 'pair-end' (2×100 cycles) on NovaSeq 6000® (Illumina®).

Sequences were aligned to the reference human genome (GRCh38/hg38) using the Burrows-Wheeler transform (BWA-MEM, 0.7.15) and data were processed in an in-house developed workflow (SeqOIA-IT platform). Variant calling (SNPs and Indels) was performed by Haplotype Caller (GATK4, v4.1.0.0) for WGS and WTS and by Mutect 2 (GATK4, v4.1.4.1) for WES. Variants were annotated using SNPeff(4.3t) and SnpSift(4.3t). CNVs were detected by Facet (v0.5.14) and WisecondorX (v1.1.5), then annotated by AnnotSV (v2.3.2). The average depth of coverage for germline WGS, tumoral WGS and WES was 46X, 111X and 233X respectively.

WT1 measurable residual disease

The quantification of *WT1* transcript was performed by real-time quantitative PCR on an ABI Prism 7900 platform (ThermoFisher) as previously described.[8] *WT1* transcript levels were normalized to the *ABL1* control gene by using the respective plasmid standards (ipsogen WT1 ProfileQuant Kit CE; Qiagen). According to ELN standards, *WT1* was considered overexpressed above background (MRD^{high}) when it was measured higher than 250 and 50 copies/10^4 *ABL1* copies in BM and PB, respectively.[9] *WT1* expression was considered as MRD^{low} when it was under these values.

SUPPLEMENTARY TABLES

Supplementary Table 1: Primers used for *UBTF* screening (from Umeda et al).[10]

Supplementary Table 2: Targeted next-generation sequencing panel (154 genes).

Supplementary Table 3: Characteristics of the 59 *UBTF*-tandem duplications. The minimal inserted region common to all but one patient (L120D8419C) is highlighted in red.

Supplementary Table 4: Clinical data of the 59 patients with *UBTF*-TD-positive AML.

Supplementary Table 5: Somatic variations identified by WES and WTS in patient #L220L8879S. Somatic variations are derived from joint analysis of constitutional and tumor sequence data.

Supplementary Table 6: Copy number variations (CNVs) identified by WGS in patient #L220L8879S.

Supplementary Table 7: Characteristics of *UBTF*-TD AML patients (18-60y) in the different cohorts. These data refer to only 57 of the 59 patients with *UBTF*-TDs (the 2 patients over 60 years of age were excluded for comparisons).

Supplementary Table 8: Characteristics of *UBTF*-TD-positive AML patients according to *FLT3*-ITD status.

SUPPLEMENTARY FIGURES

Supplementary Figure 1: Study flow chart.

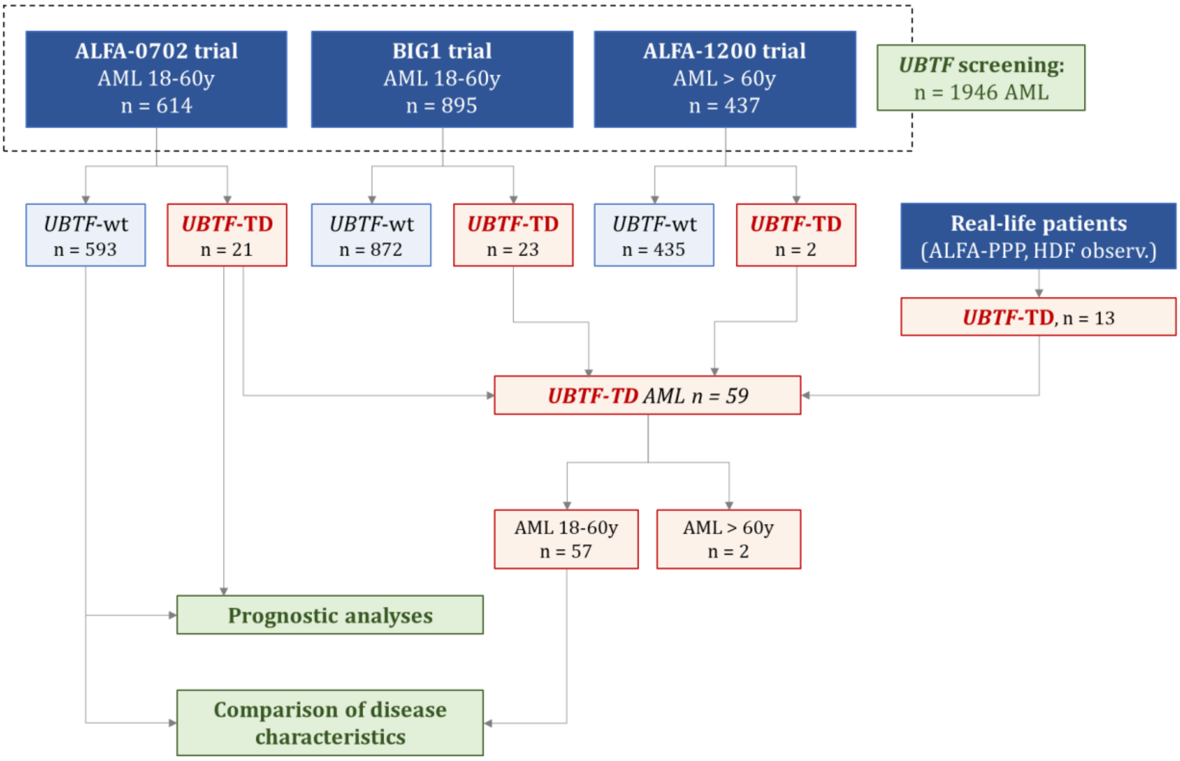

Supplementary Figure 2: WGS, WES and WTS results from patient #L220L8879S. This patient was shown to harbor 10 bp deletion followed by a large duplication (598 bp) spanning exons 12 to 14 finally leading to an exon13-exon13 fused RNA transcript. (A) Integrative Genomics Viewer (IGV) visualization of WGS showing the *UBTF* soft-clipped reads in exons 12 and 14. (B) IGV visualization of WES and WTS showing the exon 13-exon 13 fusion. (C) Summary scheme.

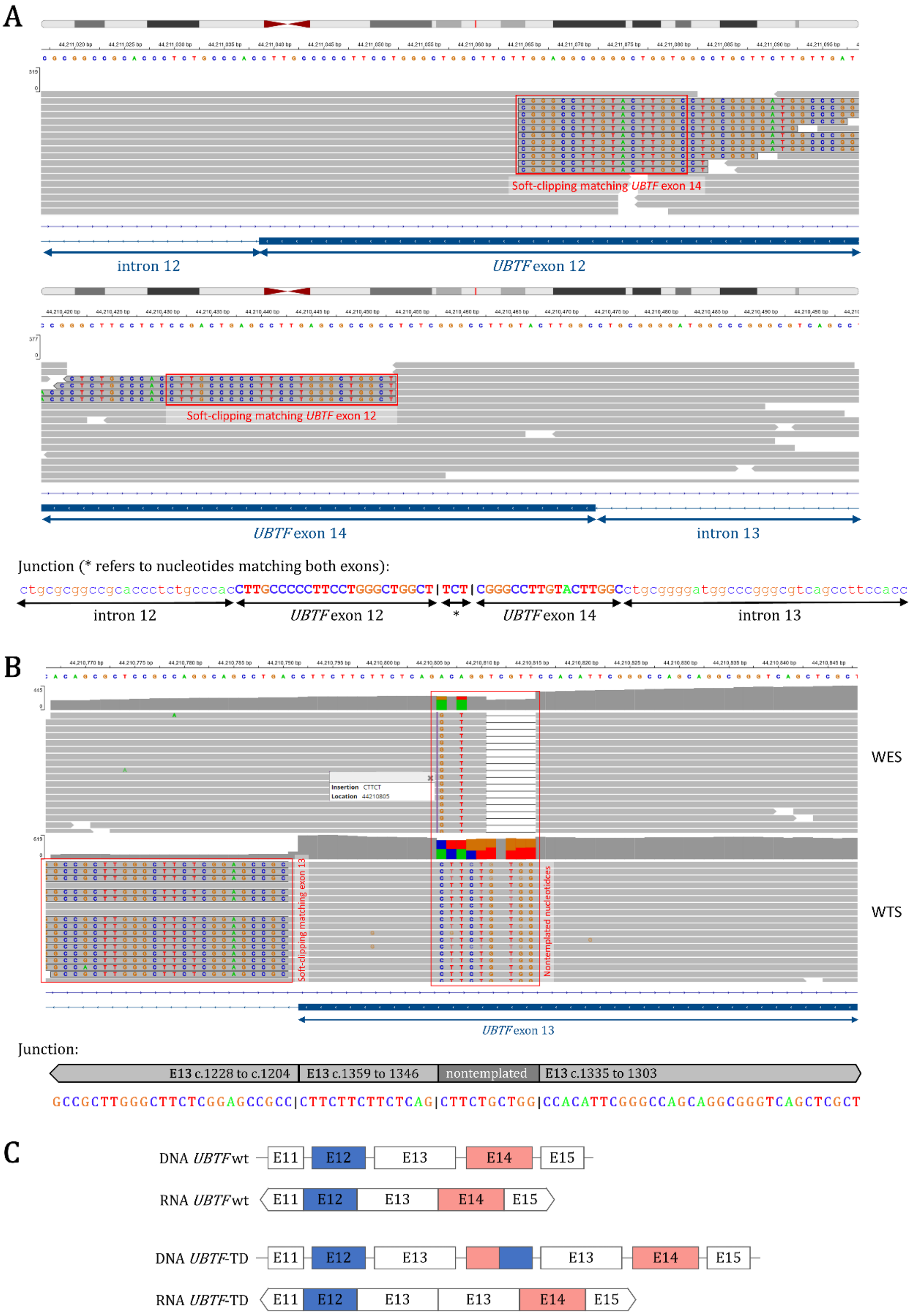

Supplementary Figure 3: Correlation of (A) WBC count and (B) bone marrow blast infiltration with the VAF of *FLT3*-ITD at *UBTF*-TD AML diagnosis. The VAF of *FLT3*-ITD was determined using the FiLT3r algorithm (Boudry et al).[3]

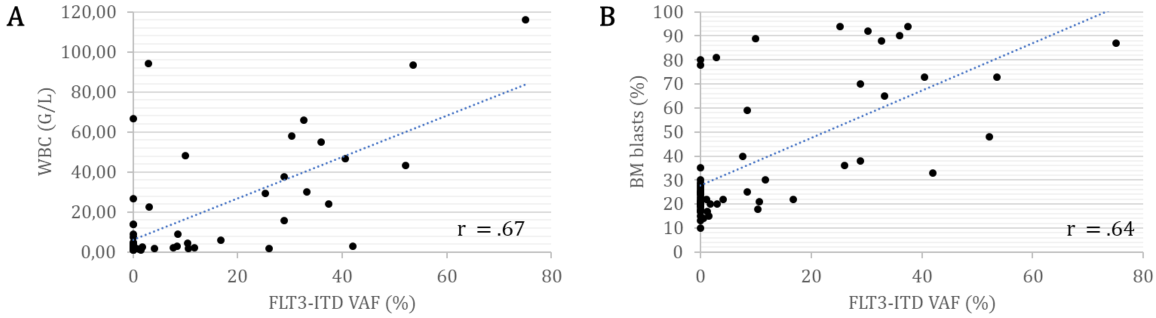

Supplementary Figure 4: Levels of *WT1* expression at diagnosis and after induction in BM. All *UBTF*-TD AML cases expressed high levels of *WT1* transcripts at diagnosis (median 446 *WT1* copies/10^4 *ABL1* copies [IQR 429-1440]). Definition of MRD^{high} was based on ELN recommendations, i.e. more than 250 *WT1* copies/10^4 *ABL1* copies in BM.[9] Patients are separated according to the response assessed by morphology after the induction treatment.

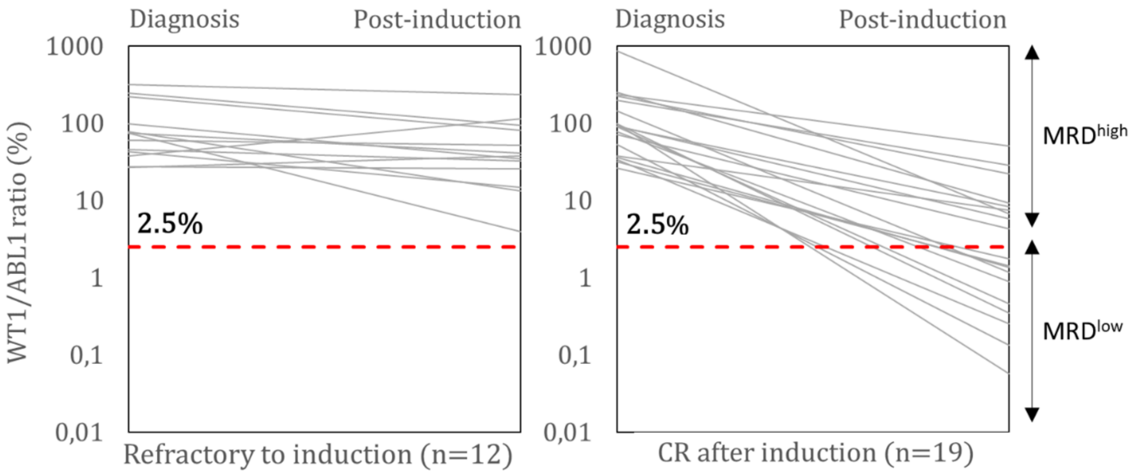

Supplementary Figure 5: Clinical impact of co-occurring mutations with *UBTF*-TD. (A) Disease-free survival (DFS) in patients achieving CR or CRp after induction and (B) overall survival (OS) un *UBTF*-TD patients according to *WT1* status. (C) DFS in patients achieving CR or CRp after induction and (B) OS un *UBTF*-TD patients according to *FLT3*-ITD status.

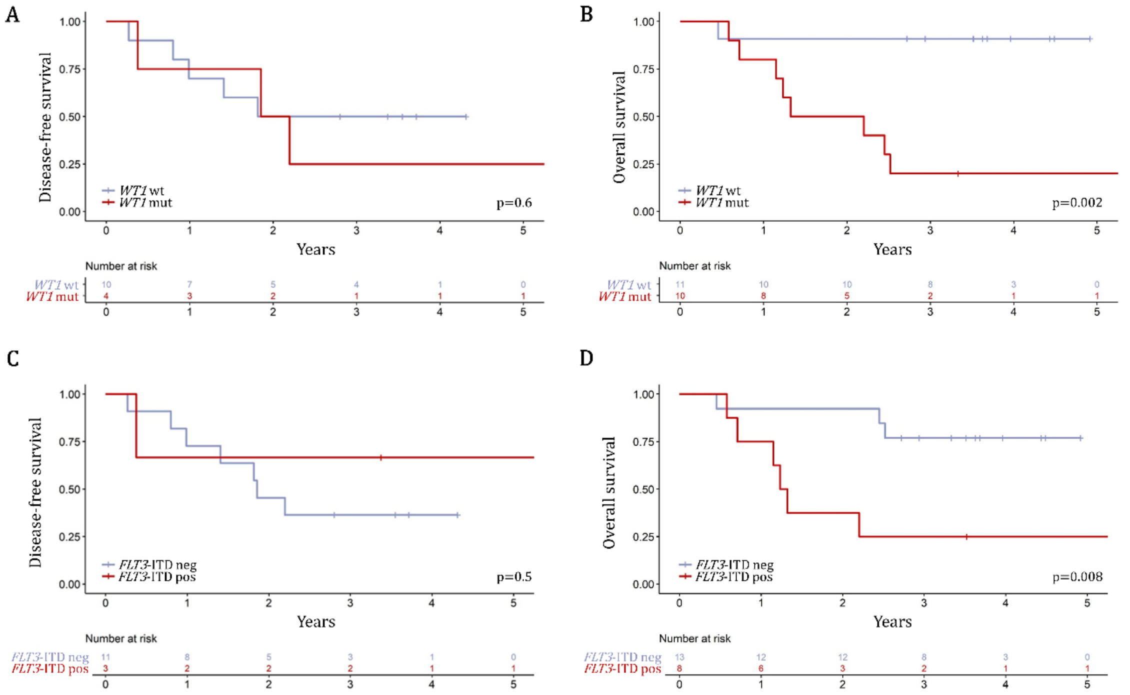

Supplementary Figure 6: Impact of HSCT in *UBTF*-TD patients in first CR/CRp. The method described by Simon and Makuch was used for graphical display of the time-dependent impact of allo-HCT on outcomes.[11] The effect of allo-HCT in first remission is represented for DFS (A) and OS from CR/CRp (B) with allo-HCT as a time-dependent covariable. Blue curves display DFS and OS from CR when the 9 patients allografted in first remission were censored at time of allo-HCT.

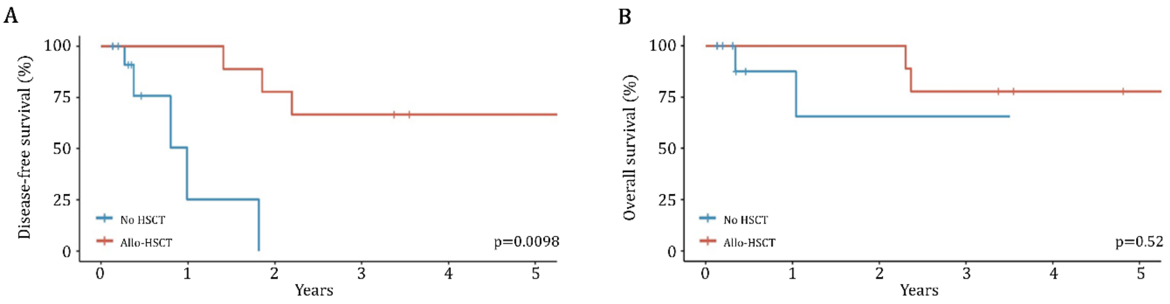

Supplementary Figure 7: Dynamics of *FLT3*-ITD and *WT1* mutations in *UBTF*-TD AML. Panel A shows the evolution of mutational status between diagnosis and relapse (n=10 patients). Panels B and C show the evolution of the cancer cell fractions (CCF) considering *UBTF*-TD as the founding lesion. The CCF is calculated as the VAF of the variant divided by the VAF of the *UBTF*-TD. Each line represents a single variant in one patient. Variants that are gained, lost or conserved at relapse are colored in red, blue and yellow respectively.

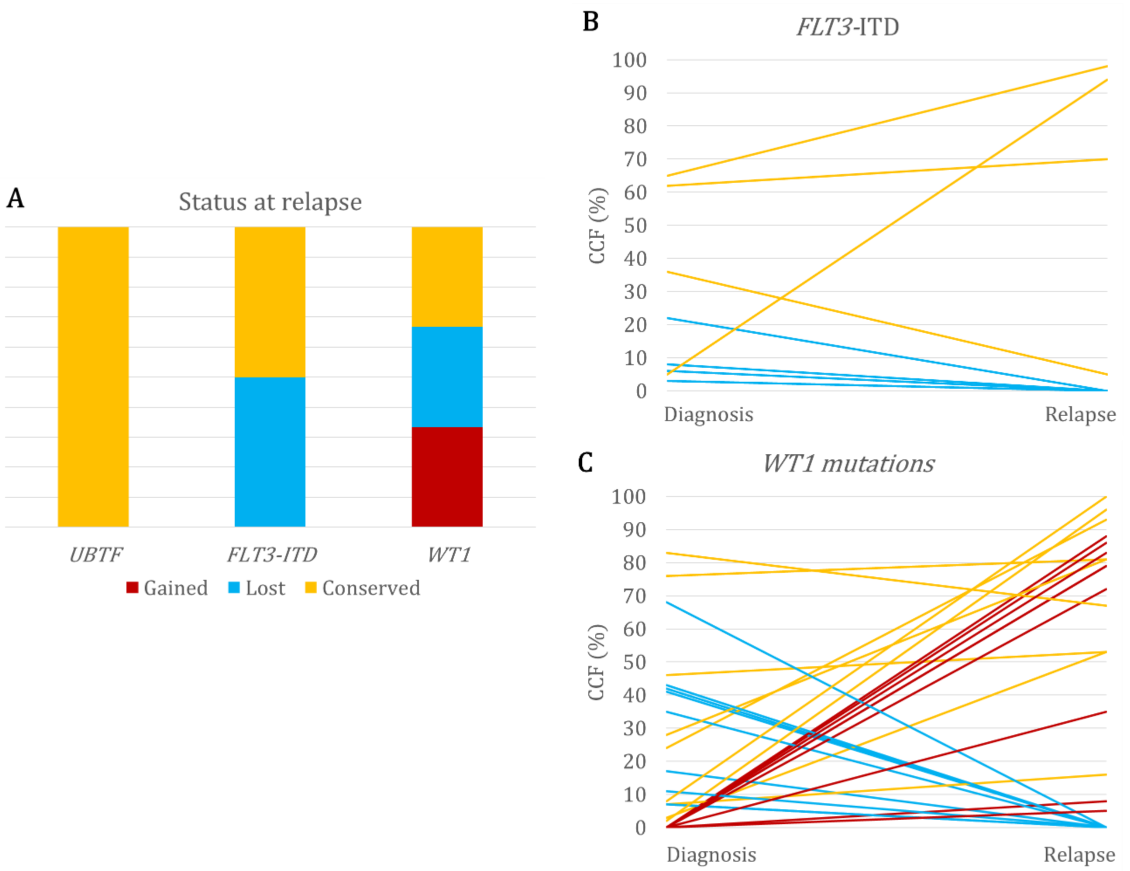

Supplementary Figure 8: Mutated cancer cell fractions in studied relapse cases (n=10). Quantification is expressed as a cancer cell fraction (CCF) considering *UBTF*-TD as the founding lesion. The CCF is calculated as the VAF of the variant divided by the VAF of the *UBTF*-TD. A sum of 2 CCFs greater than 100% suggests their coexistence within the same clone.

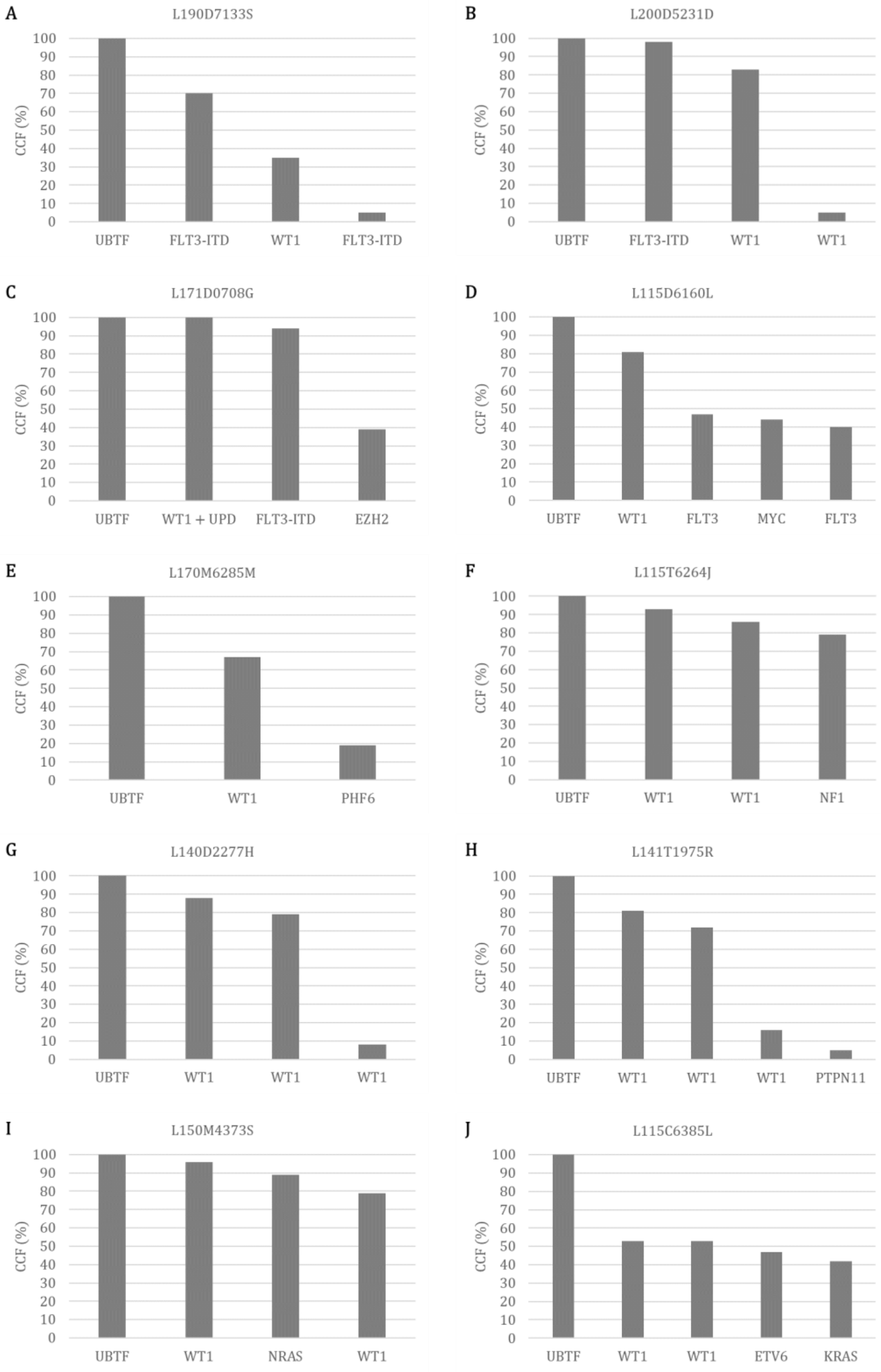

Supplementary Figure 9: Clonal evolution in patient #L190D7133S. The 2 relapse episodes occurred after treatment with ICT and quizartinib, respectively.

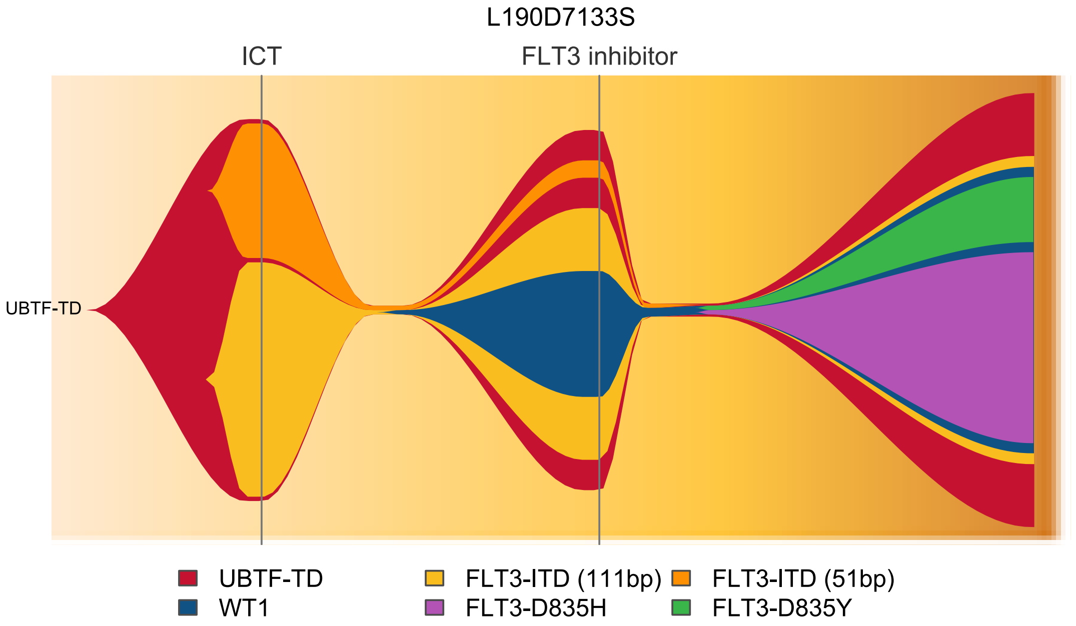

REFERENCES

1. Benjamin D, Sato T, Cibulskis K, et al. Calling Somatic SNVs and Indels with Mutect2. *bioRxiv*. 2019;861054.

2. Lai Z, Markovets A, Ahdesmaki M, et al. VarDict: a novel and versatile variant caller for next-generation sequencing in cancer research. *Nucleic Acids Res.* 2016;44(11):e108.

3. Boudry A, Darmon S, Duployez N, et al. Frugal alignment-free identification of FLT3-internal tandem duplications with FiLT3r. *BMC Bioinformatics*. 2022;23(1):448.

4. Ogino S, Gulley ML, den Dunnen JT, Wilson RB. Standard Mutation Nomenclature in Molecular Diagnostics. *J. Mol. Diagn. JMD*. 2007;9(1):1–6.

5. Miller CA, McMichael J, Dang HX, et al. Visualizing tumor evolution with the fishplot package for R. *BMC Genomics*. 2016;17(1):880.

6. Fenwarth L, Thomas X, de Botton S, et al. A personalized approach to guide allogeneic stem cell transplantation in younger adults with acute myeloid leukemia. *Blood*. 2021;137(4):524–532.

7. Itzykson R, Fournier E, Berthon C, et al. Genetic identification of patients with AML older than 60 years achieving long-term survival with intensive chemotherapy. *Blood*. 2021;138(7):507–519.

8. Lambert J, Lambert J, Thomas X, et al. Early detection of WT1 measurable residual disease identifies high-risk patients, independent of transplantation in AML. *Blood Adv.* 2021;5(23):5258–5268.

9. Cilloni D, Renneville A, Hermitte F, et al. Real-Time Quantitative Polymerase Chain Reaction Detection of Minimal Residual Disease by Standardized WT1 Assay to Enhance Risk Stratification in Acute Myeloid Leukemia: A European LeukemiaNet Study. *J. Clin. Oncol.* 2009;27(31):5195–5201.

10. Umeda M, Ma J, Huang BJ, et al. Integrated Genomic Analysis Identifies *UBTF* Tandem Duplications as a Recurrent Lesion in Pediatric Acute Myeloid Leukemia. *Blood Cancer Discov.* 2022;3(3):194–207.

11. Simon R, Makuch RW. A non-parametric graphical representation of the relationship between survival and the occurrence of an event: application to responder versus non-responder bias. *Stat. Med.* 1984;3(1):35–44.
