# Supplementary material for: UBTF tandem duplications define a distinct subtype of adult de novo acute myeloid leukemia
Source: Leukemia. 2023 Apr 21;37(6):1245–53. doi: 10.1038/s41375-023-01906-z (PMC10244165; doi:10.1038/s41375-023-01906-z)
Supplement: Supplementary file 2 — Supplemental tables [file 41375_2023_1906_MOESM2_ESM.pdf]

Supplementary Table 1: Primers used for *UBTF* screening (from Umeda et al).
Supplementary Table 2: Targeted next-generation sequencing panel (154 genes).
Supplementary Table 3: Characteristics of the 59 *UBTF* -tandem duplications. The minimal inserted region common to all but one patient (L120D8419C) is highlighted in red.
Supplementary Table 4: Clinical data of the 59 patients with *UBTF* -TD-positive AML.
Supplementary Table 5: Somatic variations identified by WES and WTS in patient #L220L8879S. Somatic variations are derived from joint analysis of constitutional and tumor sequence data.
Supplementary Table 6: Copy number variations (CNVs) identified by WGS in patient #L220L8879S.
Supplementary Table 7: Characteristics of *UBTF* -TD AML patients (18-60y) in the different cohorts. These data refer to only 57 of the 59 patients with UBTF-TDs (the 2 patients over 60 years of age were excluded for comparisons).
Supplementary Table 8: Characteristics of *UBTF* -TD-positive AML patients according to *FLT3* -ITD status.

Supplementary Table 1: Primers used for *UBTF* screening (from Umeda et al).

Primers	Sequence 5’-3’	Amplicon size (bp)
UBTF_Forward	CCA AGA AGC CAG CCC AGG AAG G	617 (wild-type)
UBTF_Reverse	GCC TCT CGG GCC TTG TAC TTG G	

Supplementary Table 2: Targeted next-generation sequencing panel (154 genes).

Gene	Transcript	Sequenced region
ABL1	NM_005157.4	exons 04-09
ANKRD26	NM_014915.2	5'UTR
ARID1A	NM_006015.4	exons 01-20
ASXL1	NM_015338.5	exons 11-12
ASXL2	NM_018263.4	exons 11-12
ATM	NM_000051.3	exons 02-63
ATRX	NM_000489.4	exons 01-35
B2M	NM_004048.2	exons 01-03
BAX	NM_138761.3	exons 01-06
BCL2	NM_000633.2	exons 02-03
BCOR	NM_001123385.1	exons 02-15
BCORL1	NM_021946.4	exons 01-12
BIRC3	NM_182962.2	exons 07-10
BRAF	NM_004333.4	exons 11,15
BRCA1	NM_007294.3	exons 02-24
BRCA2	NM_000059.3	exons 02-27
BTK	NM_000061.2	exons 02-19
CALR	NM_004343.3	exon 09
CARD11	NM_032415.5	exons 04-09
CBL	NM_005188.3	exons 08-09
CCND1	NM_053056.2	exons 01-05
CD274	NM_001267706.1	exons 02-07
CD28	NM_006139.3	exons 01-04
CD58	NM_001779.2	exons 01-06
CD79A	NM_001783.3	exons 04-05
CD79B	NM_000626.3	exons 05-06
CDKN2A	NM_058195.3	exons 01-03
CEBPA	NM_004364.4	exon 01
CHEK2	NM_001005735.1	exons 02-15
CIITA	NM_000246.3	exons 01-19
CREBBP	NM_004380.2	exons 01-31
CRLF2	NM_022148.3	exon 06
CSF2RA	NM_001161530.1	exons 03-14
CSF2RB	NM_000395.2	exons 02-14
CSF3R	NM_156039.3	exons 14-17
CUX1	NM_181552.3	exons 01-24
CXCR4	NM_003467.2	exons 01-02
DDX3X	NM_001356.4	exons 01-17
DDX41	NM_016222.3	exons 01-17
DHX15	NM_001358.2	exon 03
DHX34	NM_014681.5	exons 02-17
DNMT3A	NM_022552.4	exons 02-23
EGLN1	NM_022051.3	exons 01-05
EGR2	NM_000399.4	exon 02

EP300	NM_001429.3	exons 01-31
EPAS1	NM_001430.5	exon 09,12
EPO	NM_000799.4	exons 01-05
EPOR	NM_000121.4	exon 08
ETNK1	NM_018638.4	exons 01-08
ETV6	NM_001987.4	exons 01-08
EZH2	NM_004456.4	exons 02-20
FBXW7	NM_033632.3	exons 02-12
FGFR1	NM_001174067.1	exons 02-19
FLT3	NM_004119.2	exons 01-24
FOXO1	NM_002015.3	exons 01-02
GATA1	NM_002049.3	exons 02-03
GATA2	NM_032638.4	exons 02-06,c.1017+532_+699
GNA13	NM_006572.5	exons 01-04
GNAS	NM_000516.5	exons 08-09
GNB1	NM_002074.4	exons 05-06
HRAS	NM_005343.3	exons 02-04
ID3	NM_002167.4	exons 01-02
IDH1	NM_005896.3	exons 03-10
IDH2	NM_002168.3	exons 01-11
IKZF1	NM_006060.5	exons 02-08
IL2RG	NM_000206.2	exon 08
IL3RA	NM_002183.3	exons 02-12
IL5RA	NM_175726.3	exons 03-12
IL7R	NM_002185.3	exon 06
IRF4	NM_002460.3	exons 02-09
ITPKB	NM_002221.3	exons 02-08
JAK1	NM_002227.3	exons 02-25
JAK2	NM_004972.3	exons 03-25
JAK3	NM_000215.3	exons 02-24
KDM6A	NM_001291415.1	exons 01-30
KIT	NM_000222.2	exons 08-11,17
KLF2	NM_016270.3	exons 01-03
KMT2A	NM_001197104.1	exons 01-36
KMT2D	NM_003482.3	exons 02-55
KRAS	NM_033360.3	exons 02-04
LUC7L2	NM_001244585.1	exons 01-11
MAP2K1	NM_002755.3	exons 02-07
MAP3K14	NM_003954.4	exons 02-16
MBD4	NM_003925.2	exons 01-08
MEF2B	NM_001145785.1	exons 02-09
MPL	NM_005373.2	exons 01-12
MSC	NM_005098.3	exon 01
MYC	NM_002467.4	exons 01-03
MYD88	NM_002468.4	exons 01-05
NF1	NM_001042492.2	exons 01-58
NFE2	NM_001136023.2	exons 02-03

NIPBL	NM_133433.3	exons 02-47
NOTCH1	NM_017617.4	exons 01-34
NOTCH2	NM_024408.3	exons 26-28,34
NPM1	NM_002520.6	exons 10-11
NRAS	NM_002524.4	exons 02-04
NSD2	NM_001042424.2	exons 02-22
PAX5	NM_016734.2	exons 01-10
PDGFRA	NM_006206.5	exons 02-23
PDGFRB	NM_002609.3	exons 02-23
PHF6	NM_001015877.1	exons 02-10
PIGA	NM_002641.3	exons 02-06
PIM1	NM_002648.3	exons 01-06
PLCG1	NM_002660.2	exons 01-32
PLCG2	NM_002661.4	exons 02-33
PPM1D	NM_003620.3	exons 01-06
PRDM1	NM_001198.3	exons 01-07
PRPF8	NM_006445.3	exons 02-43
PTEN	NM_000314.6	exons 01-09
PTPN11	NM_002834.4	exons 01-15
PTPRD	NM_002839.3	exons 12-46
RAD21	NM_006265.2	exons 02-14
RB1	NM_000321.2	exons 01-27
RHOA	NM_001664.3	exons 02-05
RIT1	NM_006912.5	exon 05
RPS15	NM_001018.4	exons 03-04
RUNX1	NM_001754.4	exons 02-09
SAMD9	NM_017654.3	exon 03
SAMD9L	NM_152703.3	exon 05
SETBP1	NM_015559.2	exons 02-06
SETD2	NM_014159.6	exons 01-21
SF3B1	NM_012433.3	exons 13-18
SH2B3	NM_005475.2	exons 02-08
SMC1A	NM_006306.3	exons 01-25
SMC3	NM_005445.3	exons 01-29
SOCS1	NM_003745.1	exon 02
SPI1	NM_003120.2	exons 01-05
SRP72	NM_006947.3	exons 01-19
SRSF2	NM_003016.4	exon 01
STAG2	NM_001042749.2	exons 03-35
STAT1	NM_007315.3	exons 03-25
STAT3	NM_003150.3	exons 02-24
STAT5A	NM_003152.3	exons 03-20
STAT5B	NM_012448.3	exons 02-19
STAT6	NM_003153.4	exons 08-14
TBL1XR1	NM_024665.5	exons 03-16
TCF3	NM_003200.3	exons 02-19
TCL1A	NM_021966.2	exons 01-03

TERC	NR_001566.1	exon 01
TERT	NM_198253.2	exons 01-16
TET2	NM_001127208.2	exons 03-11
TNFAIP3	NM_006290.3	exons 02-09
TNFRSF14	NM_003820.3	exons 01-08
TP53	NM_001126112.2	exons 02-11
TYK2	NM_003331.4	exons 03-25
U2AF1	NM_006758.2	exons 02,06
UBA1	NM_003334.3	exon 03
UBTF	NM_014233.3	exons 10-15
VAV1	NM_005428.3	exons 01-27
VHL	NM_000551.4	exons 01-03
WT1	NM_024426.4	exons 01-10
XPO1	NM_003400.3	exons 12-18
ZBTB7A	NM_015898.3	exons 02-03
ZRSR2	NM_005089.3	exons 01-11

Supplementary Table 3: Characteristics of the 59 *UBTF* -tandem duplications. The minimal inserted region common to all but one patient (L120D8419C) is highlighted in red.

UPN	Gene(Transcript)	Mutation (c.hgvs)	splicing_hypo	Mutation (p.hgvs)	deleted nucleotides	deleted amino-acid	inserted nucleotides	inserted amino-acids	total duplicati
L201B37431	UBTF (NM_014233.3)	c.1335_1340delins947	YES	n.a.	c.1335_1340del	W445_D447	CCTGTCTGAGAAGAAGAAG	n.a.	941
L220L8879S	UBTF (NM_014233.3)	c.1336_1345delins608	Confirmed	p.N446_S449delins52	c.1336_1345	N446_S449	CCAGCAGAAGCTGAGAAGA	PAEAEKKKGGSEKPKRPVSAMFIFS	598
L221B25032	UBTF (NM_014233.3)	c.1509_1510ins573	YES	n.a.	.	.	CGGTTCCAGGGGCTGCAGC	n.a.	573
L115C6385L	UBTF (NM_014233.3)	c.1335_1341delins546	YES	n.a.	c.1335_1341	W445_D447	TCTGTCTGAGAAGAAGAAG	n.a.	539
L115D6160L	UBTF (NM_014233.3)	c.1335_1340delins511	YES	n.a.	c.1335_1340	W445_D447	CCTGTCTGAGAAGAAGAAG	n.a.	505
L115P9195G	UBTF (NM_014233.3)	c.1335_1336ins495	YES	n.a.	.	.	CCTAGCCTTCCCGGGGGCG	n.a.	495
L180L6707M	UBTF (NM_014233.3)	c.1333_1340delins296	YES	n.a.	c.1333_1340	W445_D447	AGCCTGTCTGAGAAGAAGA	n.a.	288
L201B38060	UBTF (NM_014233.3)	c.1335_1336ins253	YES	n.a.	.	.	CCGTCGAAACCAGCCCAGG	n.a.	253
L221T6545N	UBTF (NM_014233.3)	c.1334_1335ins174	no	p.M444_W445ins58	.	.	CCTTGGCGACACCCACGGA	CLGDTHGPRPSAPGQGGSEKPKRP	174
L171D0708G	UBTF (NM_014233.3)	c.1336_1339delins171	YES	n.a.	c.1336_1339	N446_D447	CCAGCAGGCCACCTGTCTG	n.a.	167
L170M6285M	UBTF (NM_014233.3)	c.1335_1336ins144	no	p.W445_N446ins48	.	.	CTGCAGGAGGAGCGGCCTG	LQEERPELSERPELSES**ELTRLLA**RM	144
L141H1068D	UBTF (NM_014233.3)	c.1336_1341delins150	no	p.N446_D447delins50	c.1336_1341	N446_D447	CTGTCTGAGAAGAAGAAGG	LSEKKKGGSEKPKRPVSAMFIFSEEK	144
L094M3795A	UBTF (NM_014233.3)	c.1336_1341delins144	no	p.N446_D447delins48	c.1336_1341	N446_D447	CCAGCAGACAGGCTGCTGT	PADRLLSEKPKRPVSAMFIFSEEKRR	138
L141T1975R	UBTF (NM_014233.3)	c.1336_1339delins114	YES	n.a.	c.1336_1339	N446_D447	CCTGCAGGCCACCTGTCTGA	n.a.	110
L221B26162	UBTF (NM_014233.3)	c.1336_1343delins89	no	p.N446_L448delins30	c.1336_1343	N446_L448	CCTTCTGAGAAGAAGAAGG	PSEKKKVRQLQEERPELSES**ELTRLL**	81
L160L6213P	UBTF (NM_014233.3)	c.1336_1337ins81	no	p.N446delins28	.	N446	TTTCTGACGGCCGTTCTCGG	ISDGRSRIRPELSES**ELTRLLA**RMWI	81
L151D4757X	UBTF (NM_014233.3)	c.1336_1337ins69	no	p.W445_N446ins23	.	.	TAGGTGGGAATCGGCCTGA	IGGNRPELSES**ELTRLLA**RMWIG	69
L115E6705G	UBTF (NM_014233.3)	c.1333_1334ins60	no	p.M444_W445ins20	.	.	CTACATCAGAGCGAGCTGA	STSERADPPVS**ELTRLLA**RM	60
L201B38251	UBTF (NM_014233.3)	c.1297_1299delins63	no	p.L432_S433ins20	c.1297_1299	.	CGCCTAAACCCCTCAGAGA	RLNPSES**ELTRLLA**RMTLNP	60
L201B27132	UBTF (NM_014233.3)	c.1332_1333ins60	no	p.M444_W445ins20	.	.	ACTCTCCGAGAGCGAGCTG	TLRERADPSES**ELTRLLA**RM	60
L221B26192	UBTF (NM_014233.3)	c.1332_1333ins57	no	p.M444_W445ins19	.	.	AGAATCGGAGACGATCACG	RIGDDHETES**ELTRLLA**RM	57
L104C7129V	UBTF (NM_014233.3)	c.1308_1309ins54	no	p.E436_L437ins18	.	.	AGC**GAGCTGACCCGCCTGC**	S**ELTRLLA**RMSLSGSESE	54
L120K9245J	UBTF (NM_014233.3)	c.1296_1297delins56	no	p.S433_E434ins18	c.1296_1297	.	TTCGGATCCCGAGAGC**GAG**	SDPES**ELTRLLA**RMWISD	54
L221B25151	UBTF (NM_014233.3)	c.1341_1342ins54	no	p.D447_L448ins18	.	.	GACGACGACGACGTCCGC**G**	DDDDVR**ELTRLLA**RMWND	54
L150S1663J	UBTF (NM_014233.3)	c.1335_1340delins60	no	p.W445_D447delins21	c.1335_1340	W445_D447	CCTGTCTGAGAAGAAGAAG	CLSEKKKTEES**ELTRLLA**RMC	54
L120B9697S	UBTF (NM_014233.3)	c.1299delins55	no	p.S433_E434ins18	c.1299	.	GGGAGGGGAGAGC**GAGCT**	GGES**ELTRLLA**RMSLSGG	54
L201B37632	UBTF (NM_014233.3)	c.1341_1342ins54	no	p.D447_L448ins18	.	.	TCTGACTCTGAGGTGGCG**G**	SDSEVA**ELTRLLA**RMWND	54
L190D7133S	UBTF (NM_014233.3)	c.1341_1342ins51	no	p.D447_L448ins17	.	.	GAGCCTGAGATCCGC**GAGC**	EPEIR**ELTRLLA**RMWND	51
L201B26492	UBTF (NM_014233.3)	c.1333_1334ins51	no	p.M444_W445ins17	.	.	CCCTCACCTCCGGGGAGAT	SLTSGEIG**ELTRLLA**RM	51
L221C7407J	UBTF (NM_014233.3)	c.1342_1343ins51	no	p.D447_L448ins17	.	.	CCCCTGAAGTGAGC**GAGCT**	PPEVS**ELTRLLA**RMWND	51
L104A9299B	UBTF (NM_014233.3)	c.1334_1341delins59	no	p.W445_D447delins20	c.1334_1341	W445_D447	CTCTAAGGGCCTCTGAGCTC	SLRASELSES**ELTRLLA**RMS	51
L221B26302	UBTF (NM_014233.3)	c.1342_1343ins51	no	p.D447_L448ins17	.	.	CCCCCGAGATTAGT**GAGCT**	PPEIS**ELTRLLA**RMWND	51
L115T6264J	UBTF (NM_014233.3)	c.1332_1333ins51	no	p.M444_W445ins17	.	.	GAAATCGATTCCGATTCCGA	EIDSDSES**ELTRLLA**RM	51
L180V3463P	UBTF (NM_014233.3)	c.1333_1334ins51	no	p.M444_W445ins17	.	.	CCCTCTCCGAGAGCTCCGA	SLSESSES**ELTRLLA**RM	51
L150M4373S	UBTF (NM_014233.3)	c.1326_1327ins51	no	p.A442_R443ins17	.	.	TCTCTCTCTCTCTCGGAGAG	SLSLSESSES**ELTRLLA**	51
L104D7176G	UBTF (NM_014233.3)	c.1301_1302ins51	no	p.S433_E434ins17	.	.	CGAGGAGAGC**GAGCTGAC**	DEES**ELTRLLA**RMWIDE	51
L115B7285F	UBTF (NM_014233.3)	c.1334_1335ins51	no	p.M444_W445ins17	.	.	CCTAGAAGGGGCCGAGGA	CLEGAEER**ELTRLLA**RM	51
L211S3057E	UBTF (NM_014233.3)	c.1334_1335ins48	no	p.M444_W445ins16	.	.	TCTAACTCCCCCTGAGAGC**G**	CLTPPES**ELTRLLA**RM	48
L120C9465A	UBTF (NM_014233.3)	c.1333_1334ins48	no	p.M444_W445ins16	.	.	CCCTTGAGGGCCCTGAGAG	SLEGPESE**LTRLLA**RM	48
L200C2697M	UBTF (NM_014233.3)	c.1326_1327ins48	no	p.A442_R443ins16	.	.	GCGCTCTCGCTCTCCGGCTC	ALSLSGSES**ELTRLLA**	48
L201B36941	UBTF (NM_014233.3)	c.1332_1333ins48	no	p.M444_W445ins16	.	.	GAGCTGGACCCCTCCGAGA	ELDPSES**ELTRLLA**RM	48
L094C1450V	UBTF (NM_014233.3)	c.1343_1344ins48	no	p.L448_S449ins16	.	.	CGATGTTGAC**GAGCTGACC**	DVD**ELTRLLA**RMWNDL	48
L130M5111A	UBTF (NM_014233.3)	c.1333_1334ins48	no	p.M444_W445ins16	.	.	CCCTAATGTCCCACGAGAG	SLMSHES**ELTRLLA**RM	48
L104G5833S	UBTF (NM_014233.3)	c.1334_1335ins48	no	p.M444_W445ins16	.	.	TCTAAGCGAGAGCGAGAGC	CLSESES**ELTRLLA**RM	48
L221L1992T	UBTF (NM_014233.3)	c.1333_1334ins48	no	p.M444_W445ins16	.	.	CGCTCCGAGAGGACGAGAG	SLREDES**ELTRLLA**RM	48
L170V9901T	UBTF (NM_014233.3)	c.1332_1333ins48	no	p.M444_W445ins16	.	.	GAATTGAATGAGAATGAGA	ELNENES**ELTRLLA**RM	48
L171M2671A	UBTF (NM_014233.3)	c.1341_1342ins48	no	p.D447_L448ins16	.	.	GTAGACGTCGAC**GAGCTGA**	VDVD**ELTRLLA**RMWND	48
L120N3834M	UBTF (NM_014233.3)	c.1333_1334ins48	no	p.M444_W445ins16	.	.	CCCTCTCCTCCTCCGAGAGC	SLSSSES**ELTRLLA**RM	48
L130R0357F	UBTF (NM_014233.3)	c.1333_1334ins48	no	p.M444_W445ins16	.	.	CCTTGAGCTCCTCCGAGAGC	SLSSSES**ELTRLLA**RM	48

L120A2324W	UBTF (NM_014233.3)	c.1334_1335ins48	no	p.M444_W445ins16	.	.	CCTTGGTGACTCCGAGAGC	CLGDSES**ELTRLLA**RM	48
L160A6655J	UBTF (NM_014233.3)	c.1332_1333ins48	no	p.M444_W445ins16	.	.	AGTCTAGGGGCAGACGAGA	SLGADES**ELTRLLA**RM	48
L220D9480S	UBTF (NM_014233.3)	c.1336_1337ins48	no	p.W445_N446ins16	.	.	TCAACGACCCCCAGAGC**GA**	INDPQS**ELTRLLA**RMW	48
L150C3424F	UBTF (NM_014233.3)	c.1333_1334ins48	no	p.M444_W445ins16	.	.	CCCTCTCCGACTCCGAGAGC	SLSDSES**ELTRLLA**RM	48
L130B5842M	UBTF (NM_014233.3)	c.1334_1335ins48	no	p.M444_W445ins16	.	.	CCTTGATGAGGACGAGAGC	CLDEDES**ELTRLLA**RM	48
L160L8204E	UBTF (NM_014233.3)	c.1333_1334ins48	no	p.M444_W445ins16	.	.	CGCTGAGCTCCTCCGAGAG	SLSSSES**ELTRLLA**RM	48
L201B37942	UBTF (NM_014233.3)	c.1335_1341delins52	no	p.W445_D447delins18	c.1335_1341	W445_D447	TCTGTCTGAGCTCTCCGAGA	CLSELSES**ELTRLLA**RMC	45
L140D2277H	UBTF (NM_014233.3)	c.1329_1330ins45	no	p.R443_M444ins15	.	.	CTAACCCTTGACGAGGAGA	LTLDEES**ELTRLLA**R	45
L120D8419C	UBTF (NM_014233.3)	c.1298_1299ins42	no	p.S433_E434ins14	.	.	GGGACTATGCCTCCGATTCA	GLCLRFNQLDLDQA	42
L200D5231D	UBTF (NM_014233.3)	c.1334_1348delins54	no	p.W445_E450delins19	c.1334_1348	W445_E450	CTCTACAGCTCTCCGAGAGC	SLQLSES**ELTRLLA**RMSLQ	39

Supplementary Table 4: Clinical data of the 59 patients with *UBTF* -TD-positive AML.

UPN	Cohort	Age at diagnosis	Age class	Sex	Karyotype	WBC (G/L)	BM blasts (%)	BM cellularity	Morphological subtype	Type of induction	Response to Induction	MRD positivit
L201B37431	BIG1	32	≤60y	F	47,XX,+8[20].NUC ISH(MLLX2)[200]	1.5	24	Decreased	not_available	CTI	CR after induction	not_done
L115C6385L	AML_HDF	27	≤60y	F	47,XX,+8[3]/46,XX[18]	0.98	13	Decreased	AML-M6	CTI	CR after salvage	Yes
L170M6285M	BIG1	34	≤60y	M	Failure (normal by CNV analysis)	1.75	21	Decreased	not_available	CTI	CR after induction	Yes
L211S3057E	ALFA-PPP	24	≤60y	F	46,XX[21]	2.78	59	Increased	AML-MRC	CTI	CR after induction	not_done
L171D0708G	BIG1	49	≤60y	M	46,XY[20]	1.98	22	Normal	AML-M6	CTI	CR after salvage	Yes
L120C9465A	ALFA-0702	23	≤60y	M	46,XY[20]	93.4	73	Normal	AML-M2	CTI	salvage failure	not_done
L104C7129V	ALFA-0702	29	≤60y	F	46,XX[21]	2.0	40	Decreased	AML-M2	CTI	CR after induction	Yes
L190D7133S	BIG1	38	≤60y	F	47,XX,+8[15]	46.7	73	Normal	AML-M2	CTI	CR after induction	No
L221L1992T	AML_HDF	20	≤60y	M	47,XY,+8[18]/46,XY[2]	not_available	22	not_available	AML-MRC	CTI	CR after induction	No
L201B26492	BIG1	27	≤60y	M	46,XY[25]	1.78	14	Increased	not_available	CTI	CR after salvage	not_done
L201B36941	BIG1	23	≤60y	M	47,XY,+8[4]/46,XY[42]	2.5	20	Increased	not_available	CTI	CR after salvage	not_done
L104A9299B	ALFA-0702	37	≤60y	F	46,XX[30]	22.7	20	Increased	AML-M2	CTI	CR after induction	Yes
L094M3795A	ALFA-0702	43	≤60y	M	46,XY[25]	37.8	70	Increased	AML-M2	CTI	salvage failure	not_done
L221B26192	BIG1	50	≤60y	F	46,XX[38]	1.05	15	Increased	not_available	CTI	CR after salvage	not_done
L221C7407J	ALFA-PPP	23	≤60y	F	46,XX[21]	6.12	22	Increased	AML-MRC	CTI	induction failure	not_done
L200C2697M	AML_HDF	25	≤60y	M	48,XY,+10,+22[5]/49,sl,+8[8]	55.0	90	Increased	AML-M1	CTI	CR after induction	No
L115P9195G	ALFA-0702	30	≤60y	M	46,XY[20]	15.75	38	Increased	unclassifiable	CTI	salvage failure	Yes
L094C1450V	ALFA-0702	44	≤60y	F	46,XX[20]	8.99	25	Increased	AML-M6	CTI	CR after induction	Yes
L130M5111A	ALFA-0702	43	≤60y	M	46,XY,del(9)(q22q34)[14]/46,XY[1]	1.88	36	Normal	AML-M2	CTI	salvage failure	Yes
L201B37942	BIG1	42	≤60y	F	47,XX,+8[3]/46,XX[24]	1.32	17	Increased	not_available	CTI	CR after induction	not_done
L115E6705G	ALFA-0702	18	≤60y	M	45,X,-Y[6]/46,XY[14]	30.0	65	Increased	AML-M2	CTI	salvage failure	Yes
L120K9245J	ALFA-0702	22	≤60y	M	47,XY,+8[26]/46,XY[4]	29.38	94	Increased	AML-M2	CTI	CR after induction	Yes
L104G5833S	ALFA-0702	39	≤60y	M	46,XY[20]	4.3	18	Increased	AML-M6	CTI	CR after induction	No
L180L6707M	BIG1	60	≤60y	F	47,XX,+8[14]/46,XX[6]	43.27	48	Normal	not_available	CTI	CR after salvage	not_done
L221B25151	BIG1	49	≤60y	F	46,XX[20]	94.28	81	Increased	not_available	CTI	salvage failure	not_done
L221T6545N	ALFA-PPP	45	≤60y	M	46,XY[20]	116.0	87	Increased	AML-M2	CTI + FLT3 inhibitor	CR after induction	not_done
L220L8879S	ALFA-PPP	41	≤60y	M	46,XY[20]	2.92	33	Increased	AML-MRC	CTI	induction failure	not_done
L201B38251	BIG1	48	≤60y	M	47,XY,+8[19]/46,XY[1]	23.97	94	Increased	not_available	CTI	CR after induction	not_done
L200D5231D	AML_HDF	20	≤60y	M	not_available (del(13q) by CNV analysis)	58.0	92	Increased	AML-M1	CTI	CR after induction	not_done
L170V9901T	BIG1	19	≤60y	M	46,XY[21]	65.9	88	Increased	not_available	CTI	CR after induction	Yes
L221B25032	BIG1	40	≤60y	F	46,XX[20]	48.2	89	Increased	not_available	CTI	salvage failure	not_done
L221B26302	BIG1	36	≤60y	F	46,XX[32]	2.08	30	Normal	not_available	CTI	CR after induction	not_done
L171M2671A	BIG1	55	≤60y	F	46,XX[25]	1.6	21	Normal	not_available	CTI	salvage failure	Yes
L160L6213P	BIG1	27	≤60y	M	47,XY,+8[3]/46,XY[17]	4.69	22	Decreased	not_available	CTI	CR after induction	not_done
L120N3834M	ALFA-0702	37	≤60y	M	46,XY[13]	2.4	15	Increased	AML-M6	CTI	CR after salvage	not_done
L120D8419C	ALFA-0702	23	≤60y	M	47,XX,+8[15]	13.76	80	Increased	AML-M2	CTI	CR after induction	No
L151D4757X	BIG1	22	≤60y	M	46,XY[20]	2.83	25	Increased	AML-M6	CTI	CR after induction	No
L115T6264J	ALFA-0702	19	≤60y	M	47,XY,+8[6]/46,XY[15]	1.36	10	Normal	AML-M6	CTI	CR after salvage	Yes
L130R0357F	ALFA-0702	29	≤60y	M	46,XY[26]	26.7	26	Increased	unclassifiable	CTI	salvage failure	Yes
L120A2324W	ALFA-0702	22	≤60y	M	46,XY[20]	2.05	23	Increased	AML-M6	CTI	CR after induction	not_done
L115B7285F	ALFA-0702	44	≤60y	M	46,XY[21]	8.97	25	Increased	AML-M2	CTI	CR after induction	not_done
L160A6655J	BIG1	55	≤60y	M	46,XY[50]	2.3	24	Normal	not_available	CTI	CR after induction	Yes
L220D9480S	ALFA-PPP	47	≤60y	M	not_available (gain(8p) by CNV analysis)	not_available	not_available	not_available	not_available	CTI	CR after induction	No
L150C3424F	ALFA-1200	61	>60y	M	46,XY[20]	3.6	17	not_available	AML-M6	CTI	CR after induction	No
L104D7176G	ALFA-0702	33	≤60y	M	46,XY[29]	66.6	78	Increased	AML-M1	CTI	CR after induction	Yes
L130B5842M	ALFA-0702	55	≤60y	F	46,XX[20]	3.6	29	Increased	AML-M2	CTI	CR after induction	No
L180V3463P	AML_HDF	22	≤60y	M	46,XY[20]	not_available	18	Increased	AML-M6	CTI	CR after induction	Yes
L201B37632	BIG1	52	≤60y	M	46,XY[20]	7.4	21	Increased	not_available	CTI	CR after induction	not_done
L141T1975R	AML_HDF	33	≤60y	M	46,XY[20]	not_available	not_available	Increased	AML-M6	CTI	salvage failure	Yes
L150M4373S	AML_HDF	56	≤60y	F	Failure (normal by CNV analysis)	not_available	not_available	not_available	AML-M6	CTI	CR after induction	not_done
L150S1663J	ALFA-1200	66	>60y	M	47,XY,+8[12]/46,XY[9]	4.5	35	not_available	not_available	CTI	salvage failure	Yes
L115D6160L	ALFA-0702	37	≤60y	M	46,XY[20]	13.8	20	Normal	AML-M6	CTI	salvage failure	Yes
L120B9697S	ALFA-0702	28	≤60y	F	47,XX,+8[12]/46,XX[3]	2.25	20	Normal	unclassifiable	CTI	CR after induction	No
L140D2277H	ALFA-0702	37	≤60y	M	46,XY[20]	1.2	19	Increased	AML-M6	CTI	CR after induction	not_done
L221B26162	BIG1	21	≤60y	F	46,XX[28]	1.41	28	Increased	not_available	CTI	CR after salvage	not_done
L141H1068D	AML_HDF	58	≤60y	M	47,XY,+8[15]/46,XY[5]	1.0	30	Normal	AML-M6	CTI	CR after salvage	Yes
L160L8204E	BIG1	22	≤60y	F	46,XX[30]	2.2	27	Normal	not_available	CTI	salvage failure	not_done
L201B38060	BIG1	50	≤60y	M	47,XY,+8[13]/46,XY[7]	2.2	19	Normal	not_available	CTI	CR after induction	not_done
L201B27132	BIG1	47	≤60y	M	46,XY[22]	3.5	17	Increased	not_available	CTI	CR after induction	not_done

Supplementary Table 5: Somatic variations identified by WES and WTS in patient #L220L8879S. Somatic variations are derived from joint analysis of constitutional and tumor sequence data.
Variant calling (SNPs and Indels) was performed by Haplotype Caller (GATK4, v4.1.0.0) for WGS and WTS and by Mutect 2 (GATK4, v4.1.4.1) for WES. Variants
VAF = allelic frequency; alt = number of reads supporting the mutated sequence; tot = total number of reads supporting the reported position.

Gene	Gene name	Location	Ensembl transcript identifier	HGVS nomenclature	ADN-Tumor VAF (alt/tot)	ARN-Tumor VAF (alt/tot)	CNV status	Cancer gene census list	Classification
UBTF	upstream binding transcription factor	17q21.31	ENST00000436088.5	c.1336_1345delins608	0.36(114/318)	0.53(134/252)	2/2	NA|NA	Pathogenic
FLT3	fms related receptor tyrosine kinase 3	13q12.2	ENST00000241453.11	c.1781_1782insTCTCAACCAGGTGACCGG	0.17(48/278)	0.46(6/13)	2/2	tier 1|oncogene	Pathogenic
WT1	WT1 transcription factor	11p13	ENST00000332351.7	c.1385G>A p.(Arg462Gln)	0.16(38/242)	NA(NA)	1/2	tier 1|oncogene,TSG,fusion	Pathogenic
GOLGA6L2	golgin A6 family like 2	15q11.2	ENST00000312015.6	c.258+2T>A	0.27(96/355)	NA(NA)	2/2	NA|NA	Likely pathogenic
GH2	growth hormone 2	17q23.3	ENST00000332800.7	c.456G>A p.(Trp152*)	0.31(104/333)	NA(NA)	2/2	NA|NA	Likely pathogenic
ARMCX2	armadillo repeat containing X-linked 2	Xq22.1	ENST00000328766.9	c.1118G>C p.(Arg373Pro)	0.54(114/210)	0.60(3/5)	1/1	NA|NA	Variant of undetermined significance
CMYA5	cardiomyopathy associated 5	5q14.1	ENST00000446378.2	c.383T>Cp.(Val128Ala)	0.24 (67/275)	NA (NA)	2/2	NA|NA	Variant of undetermined significance
CNGA4	cyclic nucleotide gated channel subunit alpha 4	11p15.4	ENST00000379936.2	c.1351C>Tp.(Arg451Trp)	0.33 (83/251)	NA (NA)	2/2	NA|NA	Variant of undetermined significance
DIP2A	disco interacting protein 2 homolog A	21q22.3	ENST00000417564.2	c.2981G>T p.(Arg994Leu)	0.29(81/277)	0.37(7/19)	2/2	NA|NA	Variant of undetermined significance
IARS2	isoleucyl-tRNA synthetase 2, mitochondrial	1q41	ENST00000366922.2	c.2774C>Gp.(Ser925Cys)	0.18 (35/191)	0.38 (30/80)	2/2	NA|NA	Variant of undetermined significance
ZFP28	ZFP28 zinc finger protein	19q13.43	ENST00000301318.7	c.1309G>Tp.(Val437Phe)	0.28 (59/213)	NA (NA)	2/2	NA|NA	Variant of undetermined significance

Supplementary Table 6: Copy number variations (CNVs) identified by WGS in patient #L220L8879S.
CNVs were detected by Facet (v0.5.14) and WisecondorX (v1.1.5), then annotated by AnnotSV (v2.3.2).

Chr	Start_genomic	End_genomic	Size (Mb)	Absolute copy number	Genotype	Status	Gene(s)	Cancer gene census list
[11]	32364187	32596617	0.2324	1	A1/B0	Loss	WT1	tier 1|oncogene,TSG,fusion

Supplementary Table 7: Characteristics of *UBTF* -TD AML patients (18-60y) in the different cohorts. These data refer to only 57 of the 59 patients with UBTF-TDs (the 2 patients over 60 years of age were excluded for comparisons).
MDS-related genes: *ASXL1, BCOR, EZH2, RUNX1, SF3B1, SRSF2, STAG2, U2AF1* , and/or *ZRSR2*
Abbreviations: BM, bone marrow; IQR, interquartile range; MRC, myelodysplastic-related changes; WBC, white blood cell count.

Parameters	UBTF-TD AML ALFA-0702 study	UBTF-TD AML BIG1 study	UBTF-TD AML HDF/PPP cohort	UBTF-TD AML All
No. of patients	21	23	13	57
Age, y (IQR)	33 (28-39)	40 (27-49)	27 (23-45)	36 (24-45)
WBC, $\times 10^9$/L (IQR)	8.9 (2.3-26.7)	2.3 (1.8-15.7)	4.5 (2.3-55.8)	3.55 (2-27.4)
BM blasts, % (IQR)	26 (20-65)	24 (20-39)	31 (22-80)	25 (20-63)
Cytogenetics				
Normal, n (%)	16/21 (76%)	15/23 (65%)	7/13 (54%)	38/57 (67%)
Trisomy 8, n (%)	4/21 (19%)	8/23 (35%)	4/13 (31%)	16/57 (28%)
Monosomy 5/del(5q), n (%)	0	0	0	0
Monosomy 7/del(7q), n (%)	0	0	0	0
Monosomy 17/del(17p), n (%)	0	0	0	0
Del(20q), n (%)	0	0	0	0
Del(12p), n (%)	0	0	0	0
Complex, n (%)	0	0	0	0
WT1 mutations, n (%)	15/21 (71%)	12/23 (52%)	9/13 (69%)	36/57 (63%)
Signaling mutations				
FLT3 -ITD, n (%)	10/21 (48%)	13/23 (57%)	7/13 (54%)	30/57 (53%)
FLT3 -TKD, n (%)	2/21 (10%)	2/23 (9%)	0	4/57 (7%)
NRAS, n (%)	6/21 (29%)	5/23 (22%)	2/13 (15%)	13/57 (23%)
KRAS, n (%)	4/21 (19%)	2/23 (9%)	1/13 (8%)	7/57 (12%)
PTPN11, n (%)	3/21 (14%)	1/23 (4%)	2/13 (15%)	6/57 (11%)
RIT1, n (%)	0	5/23 (22%)	1/13 (8%)	6/57 (11%)
DNA-methylation gene mutations				
DNMT3A, n (%)	2/21 (10%)	1/23 (4%)	0	3/57 (5%)
TET2, n (%)	1/21 (5%)	3/23 (13%)	1/13 (8%)	5/57 (9%)
IDH1, n (%)	0	1/23 (4%)	2/13 (15%)	3/57 (5%)
IDH2, n (%)	1/21 (5%)	1/23 (4%)	1/13 (8%)	3/57 (5%)
NPM1 mutations, n (%)	0	0	0	0
CEBPA double mutations, n (%)	0	0	0	0
TP53 mutations, n (%)	0	0	0	0
MDS-related gene mutations*, n (%)	0	2/23 (9%)	3/13 (23%)	5/57 (9%)

Supplementary Table 8: Characteristics of *UBTF* -TD-positive AML patients according to *FLT3* -ITD status.

Abbreviations: BM, bone marrow; MRC, myelodysplastic-related changes; WBC, white blood cell count,

<table>
<tr><th>Parameters</th><th>FLT3 -ITD negative</th><th>FLT3 -ITD positive <20%</th><th>FLT3 -ITD positive ≥ 20%</th></tr>
<tr><td>WBC, $\times10^9$/L (IQR)</td><td>2.4 (1.6-4.69)</td><td>2.5 (1.88-7.55)</td><td>40.53 (25.32-57.25)</td></tr>
<tr><td>BM blasts, % (IQR)</td><td>22.5 (19-26.75)</td><td>22 (19.5-32.5)</td><td>73 (52.25-89.5)</td></tr>
<tr><td>BM morphology, n(%)</td><td></td><td></td><td></td></tr>
<tr><td>Available, n</td><td colspan="2">24</td><td>10</td></tr>
<tr><td>M1/M2, n (%)</td><td colspan="2">6 (25%)</td><td>9 (90%)</td></tr>
<tr><td>M6, n (%)</td><td colspan="2">15 (62%)</td><td>0</td></tr>
<tr><td>MRC, n (%)</td><td colspan="2">3 (13%)</td><td>1 (10%)</td></tr>
</table>
